# A Butyrate-Producing Probiotic, *Clostridium butyricum* DMZ-SG06, Improves Growth Performance, Immune Function and Gut Microbiota in Largemouth Bass (*Micropterus salmoides*)

**DOI:** 10.3390/microorganisms14091931

**Published:** 2026-09-01

**Authors:** Sizhu Pan, Wei Zheng, Zhimei Feng, Zhanhong Ouyang, Mingchen Ma, Luying Huang, Lulu Wang, Xitao Wang, Chunshan Quan

**Affiliations:** 1 Department of Bioengineering, College of Life Science, Dalian Minzu University, Dalian 116600, China; 2 Key Laboratory of Biotechnology and Bioresources Utilization of Ministry of Education, College of Life Science, Dalian Minzu University, Dalian 116600, China

**Keywords:** *Clostridium butyricum*, largemouth bass, intestinal health, antioxidant capacity, immune regulation, gut microbiota

## Abstract

Intensive aquaculture and antibiotic overuse have rendered largemouth bass (*Micropterus salmoides*) prone to frequent mass mortalities and economic losses. Probiotics are promising antibiotic alternatives for improving intestinal health and immunity. This study evaluated a novel butyrate-producing strain *Clostridium butyricum* DMZ-SG06 to elucidate its growth-promoting and immunomodulatory mechanisms. Three dietary treatments were used: control (Con, basal diet), T1 (basal diet supplemented with 1 g *C. butyricum* powder containing 5 × 10^8^ CFU per 10 g diet), and T2 (basal diet supplemented with 1 mL *C. butyricum* suspension containing 5 × 10^8^ CFU per 10 g diet). A total of 270 healthy juvenile largemouth bass (5.63 ± 0.03 g) were randomly allocated to three groups (90 fish per group), with each group divided into three replicate tanks (60 × 40 × 34 cm) (30 fish per tank). Fish were fed the corresponding diets to apparent satiation for a 60-day rearing period. Results showed that T2 increased weight gain rate (WGR) by 11.35% compared with control (*p* < 0.05, 213.90 ± 12.30% vs. 192.10 ± 10.20%) and significantly improved the specific growth rate (SGR, *p* < 0.05), with T2 performing best. Probiotic treatments markedly improved intestinal morphology, nonspecific immune indices, and antioxidant status (*p* < 0.05), with T2 exerting the most prominent effects. *C. butyricum* DMZ-SG06 significantly reduced the levels of pro-inflammatory cytokines. For instance, TNF-α levels in T2 decreased 1.39-fold compared with the control group (128.57 ± 4.97 pg/g vs. 178.67 ± 7.80 pg/g, *p* < 0.05). Meanwhile, the levels of anti-inflammatory cytokines were upregulated. For example, IL-10 levels in T2 increased 1.32-fold relative to the control group (321.89 ± 15.03 pg/g vs. 244.31 ± 7.57 pg/g, *p* < 0.05). Metagenomic analysis revealed reduced *Acinetobacter* abundance, enriched beneficial genera (*Lactobacillus*, *Parabacteroides*, *p* < 0.05), and enhanced microbial functions related to the phosphotransferase system and galactose metabolism (*p* < 0.05). In conclusion, *C. butyricum* DMZ-SG06 promotes largemouth bass growth and intestinal health via butyrate metabolism, immune modulation, and microbiota remodeling. Notably, the liquid bacterial suspension formulation exerts a more significant effect on enhancing the fish’s growth performance and intestinal health than the powder counterpart, supporting its application as a safe probiotic in antibiotic-free aquaculture.

## 1. Introduction

The largemouth bass (*Micropterus salmoides*) is a commercially valuable freshwater fish species widely cultivated for its rapid growth, tender flesh, and high nutritional quality [1]. Since its introduction to China in the late 20th century, it has become a dominant species in freshwater aquaculture [2]. However, the intensification of farming practices has led to deteriorating water quality, elevated pathogen pressure, and suppressed immune functions, resulting in frequent outbreaks of intestinal diseases such as bacterial enteritis and nocardiosis [3,4]. Although antibiotics have been widely used to control these infections, their long-term application has induced antibiotic resistance, environmental contamination, and safety concerns in aquaculture products [5]. Previous studies have reported an increasing prevalence of antimicrobial resistance in pathogenic bacteria isolated from largemouth bass aquaculture regions [6,7]. Therefore, identifying safe, sustainable, and effective alternatives to antibiotics is essential for the healthy development of the largemouth bass industry.

Probiotics have emerged as promising candidates to replace antibiotics due to their ability to enhance growth, improve intestinal integrity, and modulate immune responses in aquatic animals [8]. Studies have demonstrated that probiotic supplementation can optimize gut microbiota composition, enhance antioxidant defense, and strengthen the mucosal barrier [9]. For example, multi-strain lactic acid bacteria supplements have been proven to enhance mucosal immune function and modulate the gut microbial community in African catfish (*Clarias gariepinus*) [10]. Dietary *Bacillus subtilis* supplementation can improve growth performance and antioxidant capacity, and help reshape the gut microbial community of Nile tilapia (*Oreochromis niloticus*) [11]. Host-derived lactic acid bacteria have been shown to improve intestinal morphology, barrier function and nonspecific immunity in common carp (*Cyprinus carpio*) [12]. Nevertheless, the inconsistent performance of certain probiotic strains under variable aquaculture conditions still constrains their large-scale application due to limited stress tolerance and metabolic diversity.

*Clostridium butyricum*, an anaerobic spore-forming bacterium, has recently attracted attention as a next-generation probiotic owing to its robust stress resistance and ability to produce short-chain fatty acids (SCFAs), particularly butyrate [13]. Butyrate serves as a critical energy source for intestinal epithelial cells and plays vital roles in maintaining mucosal integrity, regulating immune signaling (e.g., NF-κB and mTOR), and sustaining microbial balance [14]. In terrestrial animals, *C. butyricum* enhances growth, antioxidant capacity [15,16], and immune performance [17,18], and similar beneficial effects have been observed in multiple aquatic species, including yellow croaker, shrimp, triploid rainbow trout and channel catfish [19,20,21,22]. However, the mechanisms by which *C. butyricum* influences gut microbiota functionality and immune homeostasis in largemouth bass remain poorly understood. Furthermore, differences in the biological activity and metabolite composition between *C. butyricum* formulations (powder vs. liquid) have not been systematically compared.

In this study, we used a high-performance *C. butyricum* strain DMZ-SG06 to evaluate the effects of two different formulations (bacterial powder and bacterial liquid) on growth performance, intestinal morphology, antioxidant status, immune response, and gut microbiota of *M. salmoides*. We further explored the mechanistic links among butyrate metabolism, microbial regulation and host immune modulation. This work aimed to decipher the “butyrate–microbiota–immunity” axis behind the probiotic function of *C. butyricum*, and provide theoretical support for applying this strain as an alternative to antibiotics in sustainable aquaculture.

## 2. Materials and Methods

### 2.1. Microbial Strains and Culture Media

Three *C. butyricum* strains (DMZ-SG06, DB-02, and DB-03) preserved in the laboratory were used in this study. Strain DMZ-SG06 was isolated from aquaculture pond sediment collected in Guangzhou (Guangdong Province, China) on 15 July 2021, and the culture has been deposited at the Guangdong Microbial Culture Collection Center (GDMCC) under accession number GDMCC 62333. Two media were prepared as follows. TSC medium: tryptose 15 g, soy peptone 5 g, yeast extract 5 g, sodium metabisulfite 1 g, ferrous ammonium citrate 1 g, and agar 15 g, made up to 1000 mL with distilled water. The basal medium was autoclaved at 121 °C for 15 min. After cooling to 50 ± 1 °C, filter-sterilized 0.5% D-cycloserine solution was supplemented (20 mL per 250 mL medium) before use. RCM: peptone 10 g, beef extract 10 g, yeast extract 3 g, glucose 5 g, soluble starch 1 g, sodium chloride 5 g, sodium acetate 3 g, L-cysteine hydrochloride 0.5 g, and agar 0.5 g, made up to 1000 mL with distilled water.

### 2.2. Screening and Performance Evaluation of C. butyricum DMZ-SG06

The three *C. butyricum* strains were activated by anaerobic incubation at 37 °C for 24 h on TSC medium, followed by transfer to RCM for an additional 24 h.

Acid production capacity was evaluated by quantifying SCFAs in the fermentation broth using gas chromatography (Shimadzu GC-2010, Kyoto, Japan) equipped with a DB-FFAP column (30 m × 0.25 mm × 0.25 µm) (Agilent, Santa Clara, CA, USA). Standard curves for butyric, acetic, and propionic acids (Macklin, Shanghai, China) were used to calculate concentrations, following the method described previously [23].

Morphological characteristics of vegetative cells and spores were examined using light microscopy (Aosvi Optical, Shenzhen, China) and scanning electron microscopy (SEM) (Hitachi, Tokyo, Japan).

Strain stability was assessed by determining viable spore counts using the plate count method after storage at 25 °C for 90 days and 40 °C for 10 days. Prior to plating, sample suspensions were heat-treated in a water bath at 80 °C for 10 min to kill vegetative cells, leaving only spores for enumeration. After spreading, all plated samples were placed into anaerobic jars, flushed with premixed gas (85% N_2_, 10% CO_2_, 5% H_2_), and then incubated in a 37 °C constant-temperature incubator. Survival rates were expressed as the percentage of viable spores relative to initial counts.

Acid and bile salt tolerance were evaluated by incubating cultures at pH 2.0 or in media supplemented with 0.25% or 0.5% bile salts, respectively. Optical density (OD_600_) was recorded to estimate bacterial growth.

Powdered culture preparation was performed at two concentrations (1.0 × 10^9^ CFU/g and 5.0 × 10^8^ CFU/g). The viability of the powders was monitored at 0, 30, 60, 90, and 120 days to determine storage stability.

All the above in vitro screening experiments for the three *C. butyricum* strains were carried out independently with three biological replicates, and all in vitro assays in this section were performed at 37 °C. These results are only used for strain screening and may not fully reflect the actual butyrate-producing performance of this strain in the fish intestine (24 ± 1 °C).

### 2.3. Whole-Genome Sequencing, Assembly, and Species Identification of C. butyricum DMZ-SG06

Whole-genome sequencing of strain DMZ-SG06 was performed using a combination of the BGI DNBSEQ platform (short-read, 350 bp paired-end library) and the PacBio Sequel platform (long-read) at the Beijing Genomics Institute (BGI, Shenzhen, China). After quality filtering, 1214 Mb of clean short-read data (262-fold sequencing depth) and 5769 Mb of PacBio subread data (1247-fold sequencing depth) were obtained. The PacBio subreads were corrected using Canu (v0.3.0), and the corrected reads were assembled using Canu and Falcon, followed by single-base polishing with Quiver (SMRT Analysis v2.3.0) and GATK (v1.6-13); the final genome assembly consisted of one circular chromosome of 3,855,706 bp with a GC content of 28.71% and one plasmid of 769,297 bp with a GC content of 28.33%.

Species identification was performed at the whole-genome level, with the average nucleotide identity (ANI) between strain DMZ-SG06 and the type strain *Clostridium butyricum* DSM 10702 calculated using the OrthoANI algorithm on the EZBioCloud platform (https://www.ezbiocloud.net/tools/ani (accessed on 15 August 2026); Table S1), and the digital DNA–DNA hybridization (dDDH) value calculated using the GGDC web server (https://ggdc.dsmz.de/) with Formula (2) (Table S2).

A circular genome map of DMZ-SG06 was generated based on the assembled genome data and annotated with COG functional classifications, and pan-genome analysis was conducted by comparing gene clusters of DMZ-SG06, one laboratory strain (DB-02), and three reference *C. butyricum* strains (NBRC-13949, DSM-10702, and CDC-51208), with the resulting Venn diagram illustrating shared and unique gene clusters among these genomes.

### 2.4. Feed Preparation

Activated *C. butyricum* DMZ-SG06 was first inoculated into 30 mL of RCM and anaerobically cultured at 37 °C for 18 h to obtain a seed culture. Subsequently, 1% of the seed culture was transferred into 500 mL of fresh RCM and incubated under the same conditions for 24 h. During fermentation, the strain proliferated as vegetative cells and consumed glucose and other nutrients to produce butyric acid, hydrogen and energy. Once nutrients were depleted at the late fermentation stage, cells entered dormancy and sporulated. The resultant fermentation broth contained mainly spores with very few residual vegetative cells, yielding a bacterial suspension with a total concentration of 5 × 10^8^ CFU/mL.

The bacterial powder was prepared by Qingdao Weilan Biotechnology Co., Ltd. (Qingdao, China). Briefly, fermentation was performed under anaerobic conditions at 37 °C in RCM for 24 h. The fermentation broth was centrifuged at 12,000× *g* for 15 min to collect bacterial pellets. The cell pellets were resuspended in cryoprotectant consisting of 7.5% skim milk powder and 7.5% sucrose, followed by freeze-drying at −70 °C for 48 h. The obtained lyophilized powder was adjusted to a final viable concentration of 5 × 10^8^ CFU/g. The viability of bacterial powder was determined immediately after lyophilization, and the viable count was re-examined after storage to guarantee bacterial activity throughout the feeding trial.

All experimental feed raw materials were purchased from Tianmen Haida Feed Co., Ltd. (Tianmen, China), Haida Group. The ingredients were ground and sieved through a 60-mesh screen. Following accurate weighing and homogeneous blending, the mixed feed materials were extruded into pellets with a diameter of 2.0 mm using a twin-screw extruder (Model BF-V, Jinan Saixin Machinery Factory, Jinan, China) at a processing temperature of 120 ± 5 °C. The extruded pellets were air-dried at ambient temperature and stored in a freezer at −20 °C prior to the feeding trial. The composition and nutrient levels of the basal diet are shown in Table 1. The contents of dry matter, crude protein, crude lipid, crude ash, crude fiber, organic matter, and gross energy in the basal diet were determined according to the corresponding Chinese national standards (GB/T). For dry matter analysis, samples were dried in an oven at 105 °C for 4 h to constant weight (GB/T 6435-2015) [24]. Crude protein content was determined by the Kjeldahl method (GB/T 6432-2018) [25]. Crude lipid content was analyzed by the ether extraction method (GB/T 6433-2018) [26]. Crude ash content was quantified by incineration at 550 °C to constant weight (GB/T 6438-2018) [27]. Crude fiber content was determined by the filtration method (GB/T 6434-2015) [28]. Organic matter (OM) content was calculated as dry matter content minus crude ash content. Gross energy (GE) was determined by oxygen bomb calorimetry using a PARR 6400 oxygen bomb calorimeter (Parr Instrument Company, Moline, IL, USA).

Three dietary treatments were formulated. The control group (Con) received the unsupplemented basal diet. The T1 diet contained basal diet supplemented with *C. butyricum* powder 5 × 10^8^ CFU/g at 1 g powder per 10 g basal diet to achieve 0.5 × 10^8^ CFU (g diet)^−1^. The T2 diet contained basal diet supplemented with *C. butyricum* suspension 5 × 10^8^ CFU/mL at 1 mL suspension per 10 g basal diet to achieve 0.5 × 10^8^ CFU (g diet)^−1^. All experimental diets were sealed and stored at −20 °C.

### 2.5. Experimental Fish and Rearing Conditions

Largemouth bass were sourced from a commercial fry hatchery in Foshan, Guangdong Province, China. Following a 7-day laboratory acclimation period, all experimental fish were subjected to a 24 h fasting treatment prior to individual weighing and body length measurement to record initial body weight (IBW) and initial body length (IBL) for every fish. In total, 270 healthy juveniles of uniform size (initial body weight: 5.63 ± 0.03 g) were randomly allocated into three dietary treatments, each containing three replicate tanks with 30 fish per tank.

Fish were maintained in tanks (60 × 40 × 34 cm) for 60 days. Feeding was performed twice daily at 7:00 and 19:00 until apparent satiation. Five hours after each feeding session, uneaten feed and feces were siphoned from each tank. The collected residual feed was gently rinsed and oven-dried to constant weight; its dry mass was subtracted from the total daily feed allotment to precisely calculate daily feed intake. Water temperature was maintained at 24 ± 1 °C, with at least two partial water exchanges per day, replacing one-third of the tank volume each time. Dissolved oxygen and pH were monitored daily and maintained at >6.0 mg/L and 7.0–7.5, respectively. Ammonia–nitrogen and nitrite were kept below 0.2 mg/L and 0.15 mg/L, respectively.

### 2.6. Sample Collection

At the end of the 60-day feeding trial, all fish were fasted for 24 h. Thirteen fish were randomly selected from each tank and anesthetized with MS-222 (tricaine methanesulfonate, Macklin, Shanghai, China) at 70 mg/L. Ten fish from these 13 individuals were used for individual measurement of growth parameters, including survival rate (SR), specific growth rate (SGR), weight gain rate (WGR), and feed conversion ratio (FCR). These ten fish were returned to the pre-selected fish group upon completion of growth measurements.

Upon completion of growth assessments, five fish were randomly selected from this pre-selected group of thirteen fish for blood collection via caudal vein puncture. Blood samples were allowed to clot at room temperature for 15 min and centrifuged at 2800 rpm for 15 min at 4 °C. The obtained serum samples were stored at −80 °C for subsequent determination of antioxidant and immune-related parameters.

Another five fish were randomly selected from the remaining eight individuals, placed on an ice tray, and immediately dissected with sterile scalpels to harvest intestinal tissues. Excised intestinal tissues were rinsed three times with pre-cooled phosphate-buffered saline (PBS). Each intestinal sample was divided into two aliquots: one aliquot was fixed in 4% paraformaldehyde for histological examination, and the other was stored at −80 °C for enzyme-linked immunosorbent assay (ELISA) and gene expression analyses. The final remaining three fish were used to collect intestinal contents for metagenomic sequencing. All experimental procedures were performed in accordance with relevant bioethical standards.

### 2.7. Growth Performance Analysis

Growth performance was evaluated at the end of the 60-day trial, with ten fish randomly selected from each tank for final sampling.

Growth indices, including weight gain rate (WGR), specific growth rate (SGR), feed conversion ratio (FCR), and feed intake (FI), and survival rate (SR), were calculated using the previously described method [29] using the following formulas:

WGR (%) = [(Final body weight − Initial body weight)/(Initial body weight)] × 100;

SGR (%/d) = [(ln Final body weight − ln Initial body weight)/Feeding days] × 100;

FCR = Feed intake/(Final body weight − Initial body weight);

FI (%) = [Total feed consumption/(Final fish weight + Initial fish weight)/2]/Feeding days × 100;

SR (%) = (number of fish survival/initial fish number) × 100.

### 2.8. Intestinal Morphology Analysis

Mid-to-distal midgut segments of largemouth bass were rinsed with PBS and fixed in 4% paraformaldehyde for at least 24 h at a sample-to-fixative ratio of 1:10. Fixed tissues were dehydrated, embedded in paraffin, and sectioned at 5 μm thickness. Sections were stained with hematoxylin and eosin (H&E) following a previously described method [30]. Villus height and crypt depth were preliminarily observed under an inverted light microscope (Nikon, Tokyo, Japan); in the present study, the term “crypt depth” refers to the mucosal recess at the base of the midgut villus folds, with unified measurement landmarks defined herein: villus height was determined from the villus apex to the base of the villus fold, while crypt depth was measured from the base of the villus fold to the deepest position of the mucosal recess. Final quantification of villus height and crypt depth was performed using ImageJ software (https://imagej.net/ij/) (National Institutes of Health, Bethesda, MD, USA).

### 2.9. Hematological and Immunological Marker Assays

Eight hematological and immunological indicators were determined using commercial assay kits (Nanjing Jiancheng Bioengineering Institute, China): reduced glutathione peroxidase (GSH-Px), catalase (CAT), superoxide dismutase (SOD), total antioxidant capacity (T-AOC), malondialdehyde (MDA), acid phosphatase (ACP), immunoglobulin M (IgM), and lysozyme (LZM). All assays were conducted according to the manufacturer’s instructions, and absorbance was measured with a microplate reader (BioTek, Winooski, VT, USA).

### 2.10. RT-qPCR and ELISA Analysis

Total RNA was extracted from approximately 200 mg of intestinal tissue using the RNAeasy™ Animal RNA Isolation Kit with Spin Column (Beyotime, Shanghai, China). RNA concentration and purity were determined using a Nano-300 spectrophotometer (Allsheng, Hangzhou, China). Complementary DNA synthesis and quantitative real-time PCR (qRT-PCR) were performed using the BeyoFast™ SYBR Green One-Step qRT-PCR Kit (Beyotime, Shanghai, China). The amplification program included an initial reverse transcription at 50 °C for 20 min, followed by 95 °C for 2 min, and 39 cycles of 95 °C for 15 s and 60 °C for 30 s. Melting curve analysis was conducted from 65 °C to 95 °C in 0.5 °C increments.

Elongation factor 1-alpha (EF-1α) served as the internal reference gene, and relative gene expression levels were calculated using the 2^−ΔΔCT^ method. The Ct values of reference gene EF-1α showed no significant differences among the Con, T1 and T2 treatment groups (*p* > 0.05) (Table S3). Primer sequences are listed in Table S4. Enzyme-linked immunosorbent assay (ELISA) kits (Ruixin Bio, Quanzhou, China) were used to quantify the concentrations of TNF-α, IL-1β, and IL-10 in intestinal tissue. All samples were stored at −80 °C prior to analysis.

### 2.11. Metagenomic Sequencing Analysis

Total microbial DNA isolated from intestinal digesta samples was extracted using the E.Z.N.A.^®^ Soil DNA Kit (Omega Bio-Tek, Norcross, GA, USA). No extraction blank controls were set up in this experiment. Metagenomic libraries were constructed with the NEBNext^®^ Ultra™ DNA Library Prep Kit for Illumina (New England Biolabs, Ipswich, MA, USA). High-throughput paired-end 150 bp (PE150) sequencing was performed on the Illumina NovaSeq platform by Majorbio Bio-Pharm Technology Co., Ltd. (Shanghai, China). Approximately 10 Gb of clean sequencing data was generated for each sample.

Raw sequencing reads were quality-filtered with Fastp software (v0.20.1), and host-derived sequences were eliminated using BWA (v0.7.17). The optimized clean reads were assembled via MEGAHIT, open reading frames (ORFs) were predicted by Prodigal, and non-redundant gene catalogs were built using CD-HIT clustering. This study adopted an assembly-based analytical workflow. The Diamond tool was utilized to conduct taxonomic and functional annotations against the NR, KEGG, COG, CAZy, CARD and VFDB databases.

For microbial community diversity analysis, the Sobs index was used to evaluate α-diversity. Principal coordinate analysis (PCoA) was applied to characterize β-diversity patterns, and analysis of similarity (ANOSIM) was performed to test statistical dissimilarities in overall community structure between groups. This analysis focused on overall community compositional patterns. Variations in taxon relative abundances were reported as observational numerical trends based on stacked bar-chart visualization, without formal statistical testing for differential abundance of individual taxa. All community and functional visualization graphs were generated using R software (version 4.3.0) and Microsoft Excel (2021).

### 2.12. Data Statistics and Analysis

Statistical analyses were performed using SPSS 26.0 (IBM, Armonk, NY, USA). The tank was defined as the experimental unit, with three replicate tanks per treatment. Following the sampling scheme in Section 2.6, measurements from 13 randomly sampled fish within each tank were averaged to produce one tank-level representative value. Three tank-level values were obtained per treatment, giving nine tank-derived data points across three experimental treatments for one-way ANOVA of each measured indicator in largemouth bass. Prior to one-way analysis of variance (ANOVA), normality of data distribution and homogeneity of variances were tested. One-way ANOVA was applied to compare differences among groups, followed by Tukey’s post hoc test for multiple comparisons. Linear and quadratic polynomial contrasts were also performed to evaluate dose–response trends. Statistical significance was set at * *p* < 0.05, ** *p* < 0.01, *** *p* < 0.001, and **** *p* < 0.0001. Data are expressed as mean ± standard error of the mean (SEM). Graphs were generated using GraphPad Prism 10.0.

## 3. Results

### 3.1. Screening and Characterization of High-Butyric-Acid-Producing C. butyricum DMZ-SG06

Among the three *Clostridium butyricum* strains preserved in our laboratory, DMZ-SG06 (detailed in Section 2.1) showed superior proliferative capacity during spore germination. DMZ-SG06 exhibited a faster germination rate than the other two strains, with significant differences at 2 h, 4 h, 8 h and 10 h of incubation (*p* < 0.05). Particularly at the late incubation stage of 10 h, the OD_600_ value of DMZ-SG06 reached 1.95, which was significantly higher than DB-02 (1.83) and DB-03 (1.78) (*p* < 0.05, Table S5). Similarly, DMZ-SG06 produced significantly higher butyrate yields than the control strains DB-02 and DB-03 during the mid-to-late incubation period of 16 h, 18 h, and 24 h (*p* < 0.05). At 24 h, the butyrate concentration of DMZ-SG06 was 1.65 mg/mL, significantly higher than DB-02 (1.33 mg/mL) and DB-03 (1.52 mg/mL) (*p* < 0.05, Table 2), which reflected its outstanding butyric acid production capacity. In addition, the strain showed advantageous accumulation of total organic acids at the early incubation stage of 4 h and 6 h (Table S6), conferring superior comprehensive acid-producing performance (*p* < 0.05). Regarding stability, DMZ-SG06 maintained over 90% viability after 90 days at 25 °C and more than 60% after 10 days at 40 °C, both markedly exceeding those of the other strains (Table S7, *p* < 0.05). Based on these superior physiological traits, DMZ-SG06 was selected for subsequent experiments.

On TSC agar, the strain formed smooth, circular, black colonies (Figure S1A). Microscopic observation revealed short rod-shaped cells occurring singly or in chains, with spores located centrally or subterminally (Figure S1B). Scanning electron microscopy confirmed smooth cell surfaces with rounded ends and clearly defined, elliptical spores (Figure S1C,D). Acid and bile tolerance assays further demonstrated the strong stress resistance capacity of this strain. After 2 h exposure under pH 2.0 conditions, the viable count of DMZ-SG06 reached 11.08 log_10_ CFU/g, which was comparable to 11.09 log_10_ CFU/g in the control group (Table S8, *p* > 0.05), indicating that the survival rate was not significantly affected. Following 12 h incubation in media containing 0.25% and 0.5% bile salts, the OD_600_ values reached 1.72 and 1.70, respectively, which were nearly equivalent to the control value of 1.72, demonstrating that the strain could maintain normal growth (Table S9, *p* > 0.05). Powder stability tests showed that preparations containing 1 × 10^9^ CFU/g and 5 × 10^8^ CFU/g retained over 80% and 75% viability, respectively, after 90 days of storage (Table S10, *p* > 0.05).

### 3.2. Genomic Features and Metabolic Potential of C. butyricum DMZ-SG06

**Figure 1:**
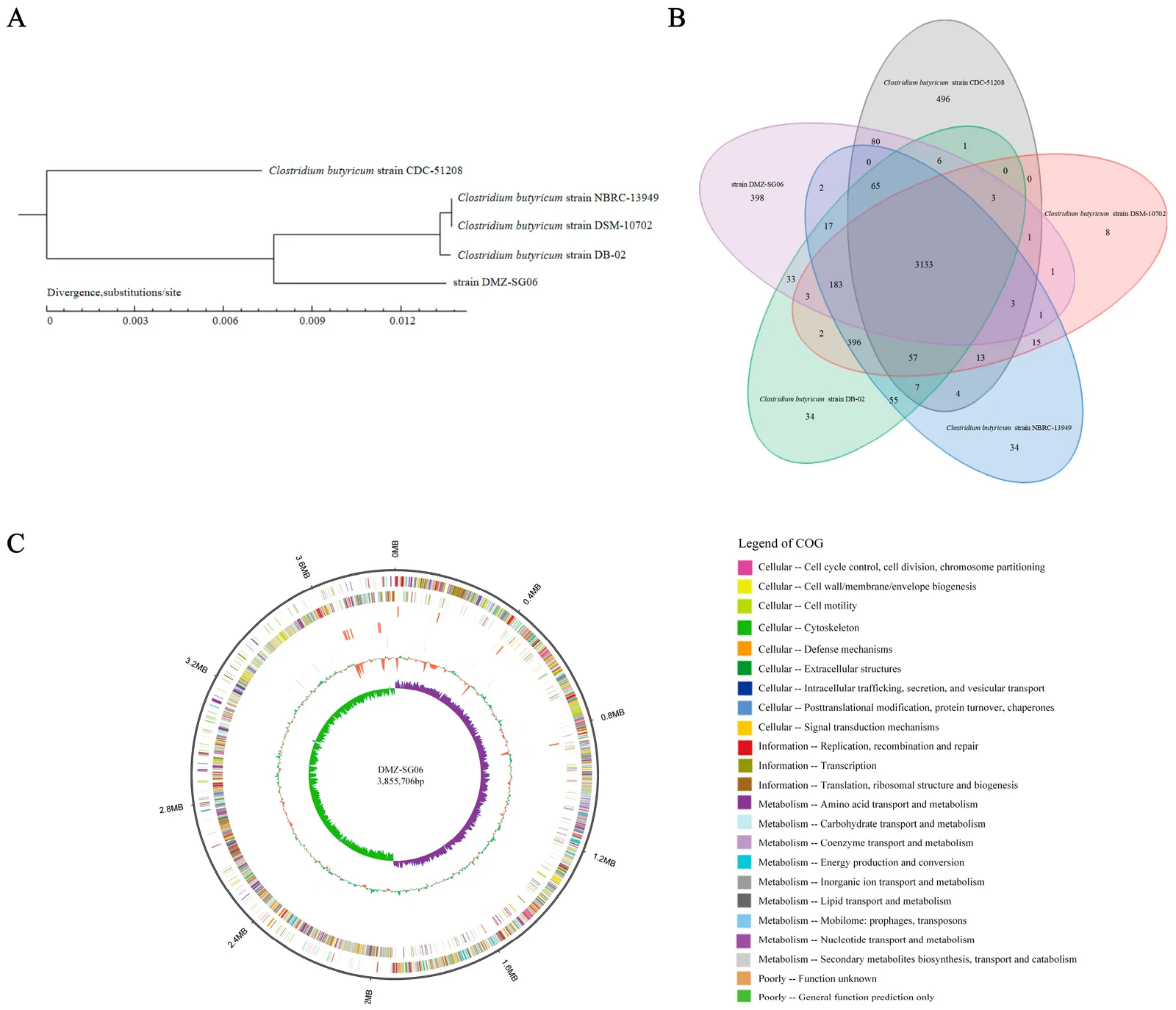
Genomic characteristic diagrams of strain DMZ-SG06. (**A**) Phylogenetic tree constructed based on the whole-genome sequence. (**B**) Venn diagram of pan-genome analysis, illustrating the overlap and uniqueness of gene clusters among *C. butyricum* strain DMZ-SG06, laboratory strain DB-02, and three type strains (*C. butyricum* CDC-51208, DSM-10702, NBRC-13949). (**C**) Circular genome map of *C. butyricum* strain DMZ-SG06 chromosome. From the outer circle to the inner circle, the outermost circle marks the genome length, and the odd and even circles represent the plus-strand and minus-strand genes, respectively. The first circle corresponds to the forward strand genes, colored according to the Cluster of Orthologous Groups (COG) classification; the second circle corresponds to the reverse strand genes, colored according to the COG classification; the third circle represents the forward strand ncRNA; the fourth circle represents the reverse strand ncRNA; the fifth circle shows the repeat sequences; the sixth circle displays the GC content distribution; and the seventh circle illustrates the GC-skew. The COG annotation color legend is shown on the right side of the figure.

Whole-genome sequencing was performed on the selected high-performing strain *C. butyricum* DMZ-SG06. The whole-genome-based species assignment revealed that strain DMZ-SG06 showed an OrthoANI value of 99.00% with the type strain *C. butyricum* DSM 10702 (Table S1), well above the 95–96% threshold for bacterial species delineation, and a dDDH value of 91.80% (Formula (2); confidence interval: 89.7–93.5%; Table S2), far exceeding the 70% threshold. These results unequivocally confirm the identification of strain DMZ-SG06 as *C. butyricum*, which was further supported by the phylogenetic placement of DMZ-SG06 within the *C. butyricum* clade (Figure 1A).

Pan-genome comparison among DMZ-SG06, DB-02, and three model strains (NBRC-13949, DSM-10702, and CDC-51208) illustrated both the shared and unique genetic compositions of these genomes (Figure 1B). DMZ-SG06 shared the largest number of core genes (3404) with NBRC-13949, whose conserved gene clusters are known to support butyrate synthesis, hydrogen production, and intestinal probiotic activity. This high overlap indicates that DMZ-SG06 retains the essential metabolic and probiotic functions characteristic of *C. butyricum*. Moreover, 398 unique gene clusters were identified in DMZ-SG06, suggesting additional genomic adaptations related to nutrient acquisition, competitive survival, and self-protection mechanisms.

The assembled genome of DMZ-SG06 contains a 3,855,706 bp circular chromosome (GC content: 28.71%) and one 769,297 bp endogenous plasmid, with a final complete genome assembly size of 4,625,003 bp (Table S11). A total of 4210 protein-coding genes were predicted, whose cumulative total length was 3,883,998 bp, accounting for 83.98% of the complete genome length. Functional annotation identified 2852 COG, 2549 GO, and 2306 KEGG entries, along with 87 tRNA, 10 5S rRNA, 10 16S rRNA, 10 23S rRNA, and 3 sRNA genes (Table S11).

The circular genome map of DMZ-SG06 displays a multilayered organization integrating gene distribution, GC content, and COG functional categories (Figure 1C). Genes associated with translation, ribosomal structure, and amino acid metabolism were widely distributed throughout the genome, suggesting a well-developed system for protein synthesis and metabolic regulation. Resistance and virulence gene analysis identified only one antibiotic resistance gene (emrA) and no high-risk virulence determinants, apart from several low-risk homologs of hemolysin and phaseolotoxin-like genes (Table S12). Genomic observations offer certain insights into the biosafety characteristics of strain DMZ-SG06.

**Figure 2:**
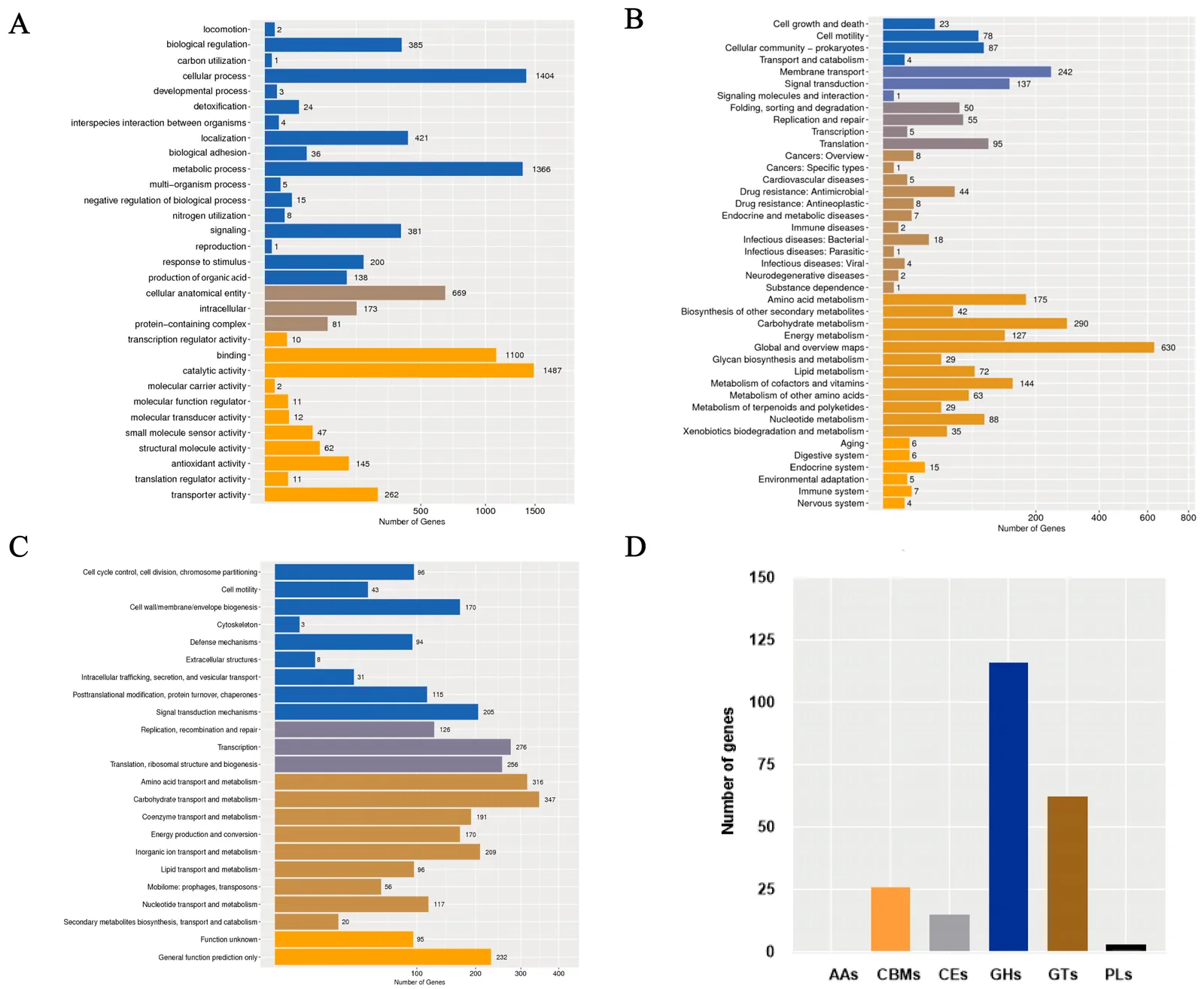
Functional annotation classification analysis of *C. butyricum* DMZ-SG06 genome. (**A**) GO functional classification. (**B**) KEGG pathway classification. (**C**) COG function classification. (**D**) CAZy function classification.

Functional annotation further revealed enrichment of genes related to catalytic activity, cellular processes, and metabolism (Figure 2A). Within the KEGG pathways, genes involved in carbohydrate metabolism (290), energy metabolism (127), and amino acid metabolism (175) were particularly abundant (Figure 2B). Butyrate biosynthesis was linked to the acetyl-CoA pathway, where key enzymes, butyryl-CoA:acetate CoA-transferase (*but*, DMZ-SG06_gene_0872, DMZ-SG06_gene_1245) and butyrate kinase (*buk*, DMZ-SG06_gene_0518, DMZ-SG06_gene_1693), were detected, supporting the strain’s strong butyrate-producing potential. In addition, genes encoding oxidoreductases and ATP synthase complexes suggest efficient energy metabolism and enhanced adaptability to hypoxic intestinal environments (Table S13).

COG functional classification (Figure 2C) indicated that carbohydrate transport and metabolism genes were the most abundant category (347 genes). Genes related to “signal transduction mechanisms” and “extracellular secretion systems” were also detected, providing a genomic foundation for host–microbe interactions through metabolic or signaling pathways. CAZy annotation (Figure 2D) revealed high abundances of glycoside hydrolases (GHs) and glycosyltransferases (GTs), emphasizing the strain’s ability to degrade and synthesize diverse polysaccharides such as plant-derived fibers, starch, and β-glucans. These enzymatic features likely enhance the strain’s colonization efficiency and ecological competitiveness in the fish intestine.

Collectively, the genomic architecture of *C. butyricum* DMZ-SG06 reflects a metabolically versatile and safe probiotic strain, genetically equipped for efficient butyrate synthesis, carbohydrate utilization, and host interaction.

### 3.3. Effects of C. butyricum DMZ-SG06 on Growth Performance and Intestinal Morphology of Largemouth Bass

After 60 days of dietary supplementation with different formulations of *C. butyricum* DMZ-SG06, significant improvements (*p* < 0.05) were observed in the body weight and weight gain rate (WGR) of largemouth bass, particularly in the liquid bacterial formulation group (T2). After the 60-day feeding period, the weight gain rate (WGR) of T2 fish was approximately 11.35% higher compared with the control group (Table 3). The specific growth rate (SGR) also increased significantly (*p* < 0.05) in the probiotic-supplemented groups, with T2 showing the highest value. No statistically significant differences in feed intake (FI) and feed conversion ratio (FCR) were observed across treatments (Table 3), although numerical variations existed among groups. Partial mortality was observed in the Con and T1 groups, with average survival rates of 96.67% and 98.89%, respectively, whereas no fish died in the T2 group with a survival rate of 100% (Table 3). These findings collectively indicate that dietary supplementation with *C. butyricum*, especially in liquid form, promotes growth and enhances feed efficiency in largemouth bass.

**Figure 3:**
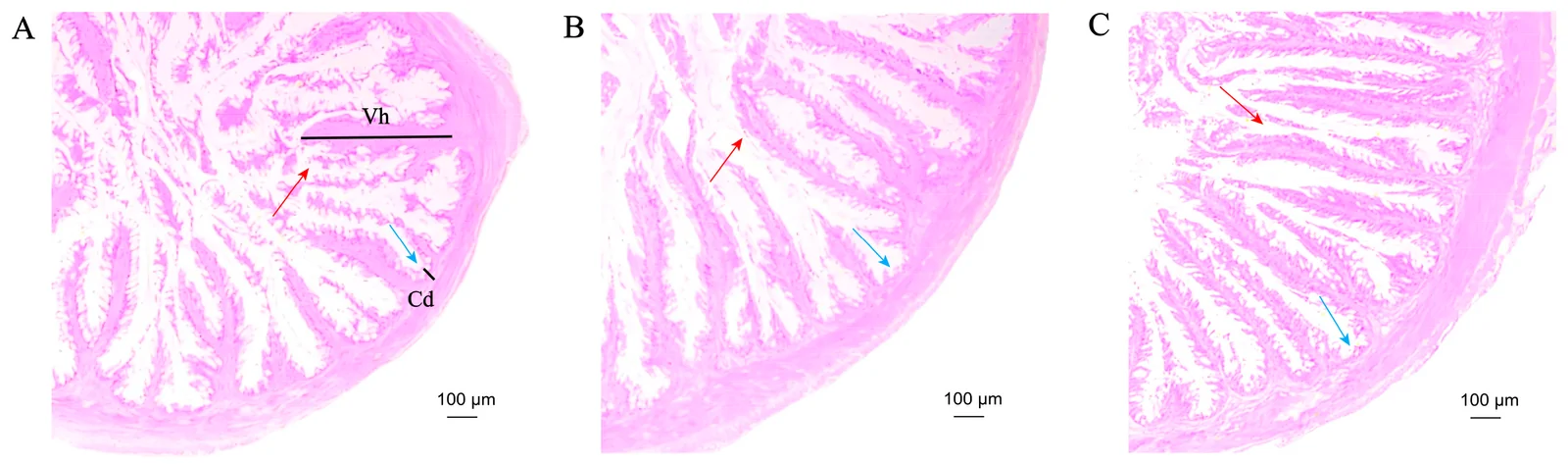
Intestinal histological observation of largemouth bass. The letters in image corners (**A**–**C**) represent Con, T1, and T2, respectively. Scale bar = 100 μm. The red arrow indicates intestinal villi fusion or atrophy, and the blue arrow indicates crypt depth or inflammatory cell infiltration. Con, T1 and T2 indicate largemouth bass fed a basal diet with 0, 1 g *C. butyricum* DMZ-SG06 powder (5 × 10^8^ CFU/g) per 10 g diet, and 1 mL *C. butyricum* DMZ-SG06 bacterial solution (5 × 10^8^ CFU/mL) per 10 g diet.

Histological analysis revealed distinct differences among treatment groups. In the control group, intestinal villi were shortened and sparsely distributed, with partial fusion or atrophy, deep crypts, and pronounced inflammatory cell infiltration (Figure 3A). As shown in Table 4, dietary supplementation with *C. butyricum* DMZ-SG06 markedly improved intestinal morphological parameters. Compared with the Con group, the T2 group exhibited a 24.74% increase in villous height (529.87 μm vs. 424.79 μm, *p* < 0.05), a 17.58% reduction in crypt depth (28.64 μm vs. 34.75 μm, *p* < 0.05), and a 51.35% elevation in the villus height-to-crypt depth ratio (VH/CD, 18.51 vs. 12.23, *p* < 0.05). In the T1 group, villi appeared longer and more regularly arranged, crypts were slightly shallower, and inflammation was reduced (Figure 3B). The T2 group exhibited the most intact intestinal architecture, characterized by dense, elongated villi, well-developed crypts, and minimal inflammatory cell presence (Figure 3C). These morphological improvements indicate that *C. butyricum* supplementation strengthens intestinal barrier integrity, promotes mucosal renewal, and reduces local inflammation in largemouth bass. The superior performance of the liquid formulation (T2) is consistent with its higher bacterial viability and more stable acid-producing activity, which together support better intestinal health and nutrient utilization.

Overall, these results confirm that *C. butyricum* DMZ-SG06, particularly in liquid form, effectively improves growth performance and intestinal morphology in largemouth bass, establishing a physiological foundation for its immunomodulatory and metabolic effects examined in subsequent sections.

### 3.4. Effects of C. butyricum DMZ-SG06 on Antioxidant Capacity and Immune Responses

Dietary supplementation with *C. butyricum* DMZ-SG06 markedly enhanced the antioxidant defense system and immune function of largemouth bass. Serum immune indices showed significant increases (*p* < 0.05) in lysozyme (LZM), acid phosphatase (ACP), and immunoglobulin M (IgM) levels in both probiotic-treated groups (T1 and T2) compared with the control (Table 5). Moreover, IgM levels were higher in the T2 group than in T1, indicating a stronger humoral immune response associated with the liquid bacterial formulation.

The activities of superoxide dismutase (SOD) and total antioxidant capacity (T-AOC) were significantly elevated (*p* < 0.05) in the probiotic-fed groups (Table 6). Higher catalase (CAT) and glutathione peroxidase (GSH-Px) activities were observed in the T2 group relative to the control (*p* < 0.05). Concurrently, malondialdehyde (MDA), an indicator of lipid peroxidation, decreased significantly (*p* < 0.05), reflecting reduced oxidative damage and improved membrane stability. Among treatments, T2 exhibited the highest antioxidant enzyme activities and the lowest MDA levels, suggesting that the liquid formulation provided more effective antioxidant protection, likely due to higher viable bacterial counts and stable metabolite release.

**Figure 4:**
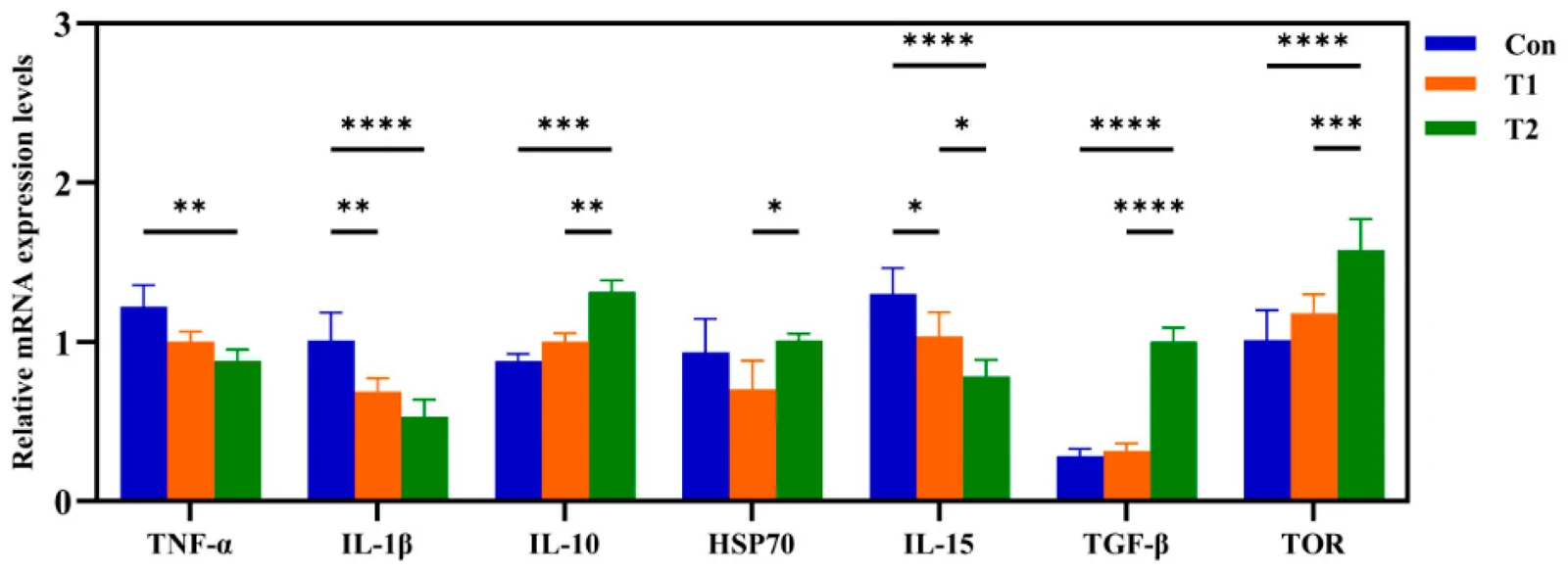
Effects of *C. butyricum* DMZ-SG06 on the relative mRNA expression of inflammatory cytokines in the intestine of largemouth bass. The relative expression of each target gene was normalized to the housekeeping gene EF-1α and calculated using the 2^−ΔΔCt^ method. TNF-α = tumor necrosis factor α; IL-1β = interleukin 1β; IL-10 = interleukin 10; HSP70 = heat shock protein 70; IL-15 = interleukin 15; TGF-β = transforming growth factor β; TOR = target of rapamycin. Data are shown as means ± SEM (*n* = 3). Con, T1 and T2 indicate largemouth bass fed a basal diet with 0, 1 g *C. butyricum* DMZ-SG06 powder (5 × 10^8^ CFU/g) per 10 g diet, and 1 mL *C. butyricum* DMZ-SG06 bacterial solution (5 × 10^8^ CFU/mL) per 10 g diet, respectively. Data with different asterisks indicate significant difference (* *p* < 0.05, ** *p* < 0.01, *** *p* < 0.001, **** *p* < 0.0001). The exact *p*-values for the pairwise comparisons shown in this figure are listed in Table S14 to ensure full transparency of the statistical results.

At the molecular level, significant downregulation of pro-inflammatory cytokines (IL-1β and IL-15) was observed in intestinal tissues of both T1 and T2 groups, while anti-inflammatory genes (IL-10 and TGF-β) and the key signaling regulator TOR were upregulated (Figure 4, *p* < 0.05). Furthermore, significant downregulation of the pro-inflammatory cytokine TNF-α relative to the control was detected in the T2 group (*p* < 0.05), demonstrating its more prominent advantage in inhibiting inflammation. These changes imply that *C. butyricum* supplementation modulates intestinal immune balance by suppressing inflammatory signaling pathways and enhancing anti-inflammatory pathways. The trends obtained from ELISA results (Table 7) were consistent with the mRNA expression data, confirming this coordinated regulatory mechanism characterized by enhanced antioxidant activity, attenuated inflammation, and improved immune response.

Overall, these results demonstrate that *C. butyricum* DMZ-SG06 enhances antioxidant defenses and immune regulation through a coordinated “antioxidant–anti-inflammatory–immune enhancement” mechanism, providing a physiological basis for subsequent mechanistic interpretation.

### 3.5. Modulation of Intestinal Microbiota Composition and Metabolic Function

**Figure 5:**
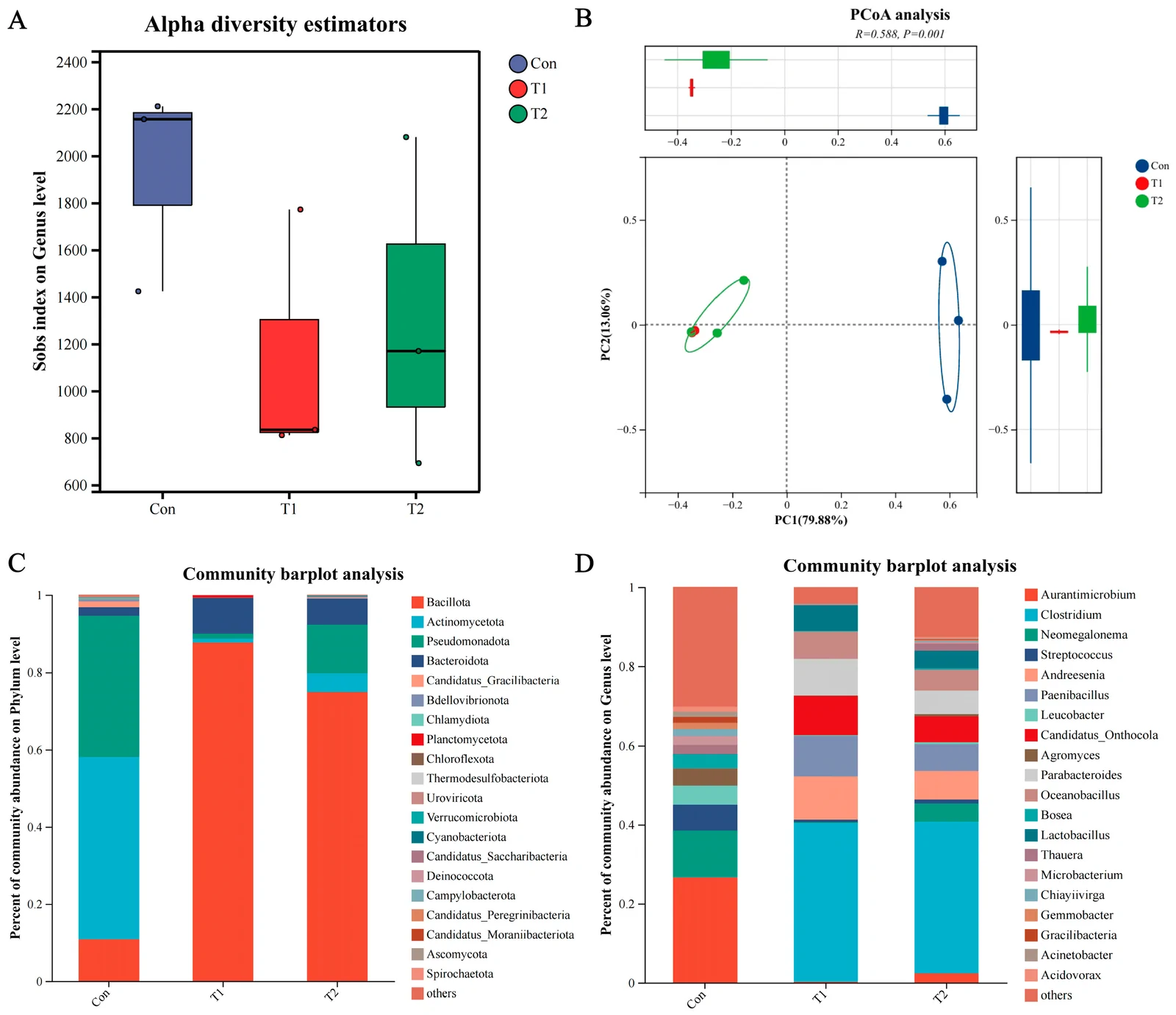
Effects of *C. butyricum* DMZ-SG06 on intestinal microbial diversity and composition. (**A**) Alpha diversity estimators (Sobs index) at the genus level. (**B**) PCoA analysis based on microbial community composition, indicating β diversity differences among groups (R = 0.588, *p* = 0.001). (**C**) Relative abundance at the bacterial phylum level in intestine. (**D**) Relative abundance at the bacterial genus level in intestine. Data are shown as means ± SEM (*n* = 3). Con, T1 and T2 indicate largemouth bass fed a basal diet with 0, 1 g *C. butyricum* DMZ-SG06 powder (5 × 10^8^ CFU/g) per 10 g diet, and 1 mL *C. butyricum* DMZ-SG06 bacterial solution (5 × 10^8^ CFU/mL) per 10 g diet.

Given the marked improvements in growth, antioxidant capacity, and immune function observed in probiotic-treated groups, further analyses were conducted to explore the underlying microbial and metabolic mechanisms. Metagenomic sequencing was therefore employed to characterize the effects of *C. butyricum* DMZ-SG06 on intestinal microbiota composition and functional profiles in largemouth bass. Metagenomic sequencing revealed that dietary supplementation with *C. butyricum* DMZ-SG06 significantly modulated the gut microbial structure and function of largemouth bass (Figure 5).

Analysis of α-diversity (Figure 5A) showed that the Sobs index significantly decreased in both T1 and T2 groups (*p* < 0.05), which reflected the reduction in observed species richness of intestinal microbiota. β-diversity analysis (Figure 5B) demonstrated clear separation between probiotic-treated groups and the control group in PCoA plots (R = 0.588, *p* = 0.001), confirming that *C. butyricum* supplementation markedly altered intestinal microbial composition. At the phylum level (Figure 5C), *Bacillota*, *Actinomycetota*, and *Pseudomonadota* dominated the intestinal microbiota, collectively accounting for more than 90% of total abundance. Following *C. butyricum* supplementation, the relative abundance of *Bacillota* increased, while the proportions of *Actinomycetota* and *Pseudomonadota* decreased. The enrichment of *Bacillota*, a major group of butyrate-producing bacteria, suggests a shift toward a more functionally stable, SCFA-producing microbial community. At the genus level (Figure 5D), there were numerical increases in the relative abundances of *Clostridium*, *Lactobacillus*, *Paenibacillus* and *Parabacteroides*, while the proportions of potentially pathogenic genera including *Pseudomonas* and *Acinetobacter* showed numerical decreases. These shifts suggest a potential improvement of intestinal anti-inflammatory capacity.

**Figure 6:**
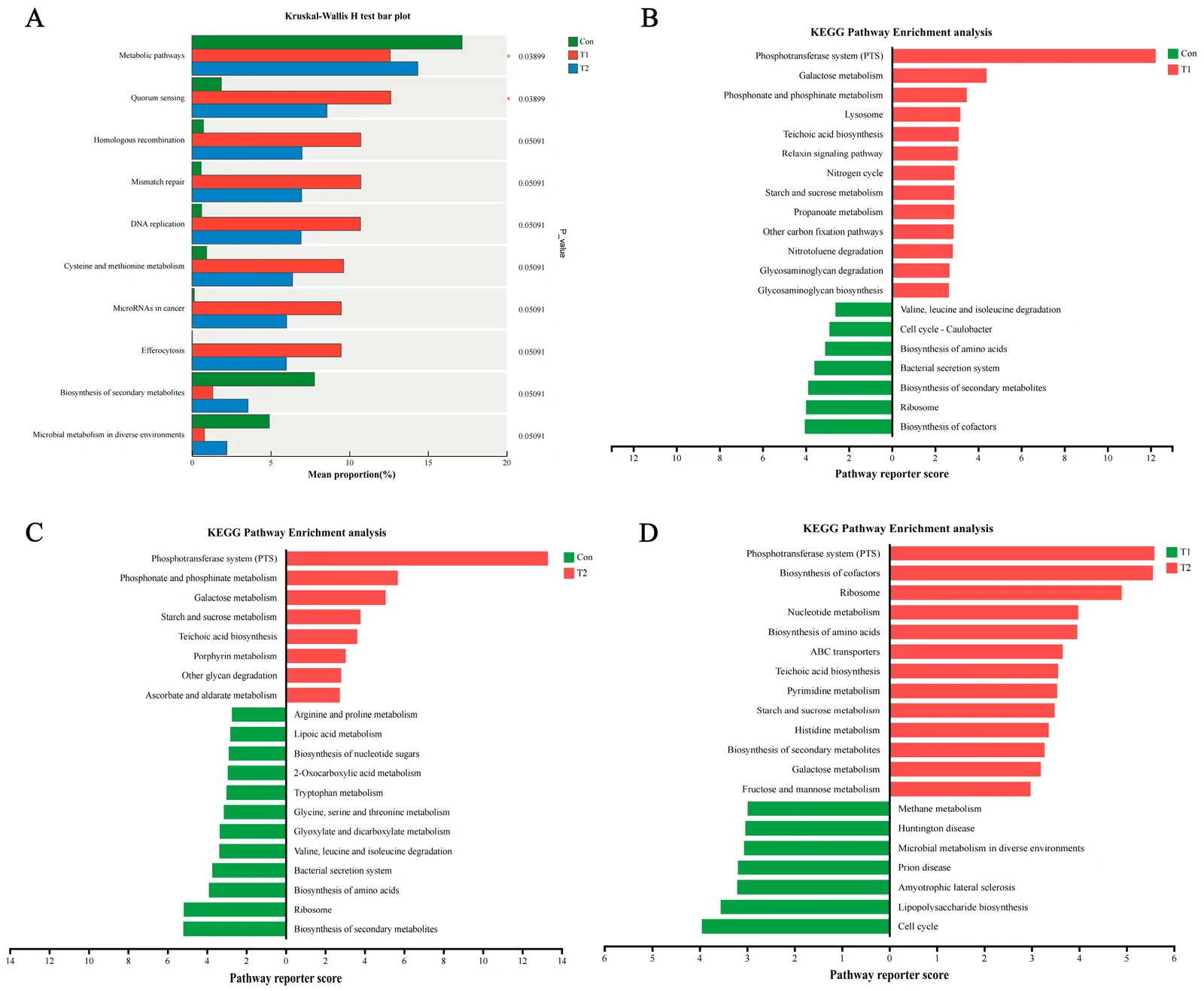
Functional analysis of intestinal microbiota modulated by *C. butyricum* DMZ-SG06. (**A**) Kruskal–Wallis H test bar plot of metabolic pathways. The asterisk denotes significant difference at *p* < 0.05. (**B**–**D**) Pathway reporter score of metabolic pathways in Con, T1 and T2 groups, respectively. Pathway reporter score: positive values (bars to the right of zero) indicate enhanced pathway activity for the corresponding group; negative values (bars to the left of zero) indicate suppressed pathway activity. Data are shown as means ± SEM (*n* = 3). Con, T1 and T2 indicate largemouth bass fed a basal diet with 0, 1 g *C. butyricum* DMZ-SG06 powder (5 × 10^8^ CFU/g) per 10 g diet, and 1 mL *C. butyricum* DMZ-SG06 bacterial solution (5 × 10^8^ CFU/mL) per 10 g diet.

Functional annotation based on the KEGG database (Figure S2) revealed that microbial metabolic capacity was substantially enhanced in both T1 and T2 groups. Pathways related to “Metabolic processes” and “Quorum sensing” were significantly enriched compared with the control (Figure 6A, *p* < 0.05), implying heightened microbial activity and interspecies communication. Enrichment in the phosphotransferase system (PTS) and galactose metabolism pathways suggested improved carbohydrate utilization, potentially increasing substrate availability for SCFA biosynthesis and supporting intestinal energy balance (Figure 6B). The liquid bacterial formulation (T2) displayed the highest enrichment across most functional modules, particularly those associated with energy metabolism, amino acid metabolism, and xenobiotic biodegradation (Figure 6C,D). These enhancements indicate a more balanced and metabolically active gut environment, contributing to improved nutrient transformation and host–microbe metabolic interactions.

Taken together, these findings demonstrate that *C. butyricum* DMZ-SG06 reshapes the intestinal microbiota toward a more beneficial, butyrate-producing configuration while enhancing microbial metabolic efficiency. The resulting increase in SCFA production and nutrient utilization provides a microbiological and metabolic foundation for the improved antioxidant capacity, immune regulation, and intestinal health observed in largemouth bass.

## 4. Discussion

This study explores potential mechanisms by which *C. butyricum* DMZ-SG06 promotes growth, antioxidant defense, immune regulation, and intestinal homeostasis in largemouth bass. By integrating physiological, biochemical, molecular, and metagenomic analyses, our results suggest that this butyrate-producing strain exerts multi-level regulatory effects that collectively improve host performance and gut health. Unlike previous studies focusing on single physiological indicators, the present work provides a correlative mechanistic framework linking butyrate metabolism, immune signaling pathways, and microbiome remodeling, thereby offering new insights into probiotic–host interactions and their potential applications in sustainable aquaculture.

Results from strain screening showed that *Clostridium butyricum* DMZ-SG06 displayed outstanding acid-producing ability, stress tolerance and stability, suggesting great adaptability for use as a probiotic. Whole-genome analysis revealed that DMZ-SG06 possesses an exceptionally rich repertoire of genes involved in carbohydrate and energy metabolism, particularly those associated with glycoside hydrolases (GHs) and glycosyltransferases (GTs). The coexistence of complete butyrate kinase and butyryl-CoA:acetate CoA-transferase pathways suggests high butyrate biosynthetic efficiency [31], consistent with the metabolic versatility characteristic of *C. butyricum* [32,33]. These genomic traits confer broad substrate adaptability and metabolic robustness, enabling efficient carbohydrate degradation and energy conversion within the fish intestine. Such capacity not only supports stable colonization under fluctuating aquaculture conditions but also ensures a continuous supply of short-chain fatty acids (SCFAs), particularly butyrate, which serves as a key molecular link between microbial metabolism and host physiology.

Improved growth performance and feed utilization efficiency have been observed in probiotic-treated fish [34,35]. Notably, liquid formulations exhibit significant advantages in biological efficacy over powder preparations [36]. As described in Section 2.4, the liquid formulation retains metabolites produced by *Clostridium butyricum* DMZ-SG06, mainly butyrate, enabling synergistic effects between butyrate and probiotic spores. In contrast, powder preparations contain only purified spores without these bioactive metabolites. This reflects the contribution of synergistic interactions between viable probiotic cells and bioactive metabolites such as butyrate, vitamins, and antimicrobial peptides [37,38]. These metabolites are known to promote intestinal epithelial cell proliferation and enzyme activities, thereby enhancing nutrient absorption. Meanwhile, increased antioxidant enzyme activities (SOD, CAT, and GSH-Px), accompanied by reduced levels of the lipid peroxidation product MDA, indicate that DMZ-SG06 strengthens the host oxidative defense system. This effect is consistent with the notion that butyrate may regulate the Keap1-Nrf2 antioxidant signaling pathway in largemouth bass [39]. It has been reported that under oxidative or electrophilic stress, Keap1 undergoes conformational modification to release Nrf2; the liberated Nrf2 translocates into the nucleus, heterodimerizes with Maf proteins, and binds to ARE motifs to initiate transcription of downstream antioxidant-responsive genes [40]. These findings highlight the importance of probiotic viability and metabolite stability in determining functional outcomes. Such formulation-dependent differences provide valuable references for optimizing probiotic preparation and delivery strategies in aquaculture.

In terms of immune regulation, *C. butyricum* DMZ-SG06 significantly modulated cytokine expression profiles by downregulating pro-inflammatory mediators (TNF-α, IL-1β, IL-15) and upregulating anti-inflammatory cytokines (IL-10, TGF-β) [41,42], thus re-establishing intestinal immune equilibrium [43,44]. These findings are consistent with the anti-inflammatory effects of sodium butyrate observed in mammals and highlight the conserved immunomodulatory function of butyrate across vertebrate species [45]. Importantly, as a core nutrient-sensing signaling hub in teleost fish, TOR integrates nutrient inputs to govern protein biosynthesis, cellular metabolic reprogramming and immune-cell homeostasis. Its signaling output can be triggered by short-chain fatty-acid metabolites such as butyrate, which coordinates the balance between somatic growth and immune responses [46]. The activation of the TOR signaling pathway observed in the present study suggests that *Clostridium butyricum* not only suppresses inflammatory responses but may also promote cellular energy metabolism and immune cell proliferation via metabolic signaling [47]. This mechanistic insight expands current understanding of fish probiotic function, moving beyond cytokine-level effects to reveal a deeper metabolic–immune regulatory axis involving the TOR pathway.

At the microbiological level, metagenomic analysis revealed that DMZ-SG06 substantially reshaped the intestinal microbiota, shifting the community from an *Actinomycetota*-dominant structure toward one enriched in *Bacillota*, a phylum comprising many butyrate-producing and lactic acid bacteria. The enrichment of *Clostridium*, *Lactobacillus*, and *Paenibacillus*, coupled with the reduction in opportunistic pathogens such as *Pseudomonas* and *Acinetobacter*, reflects the establishment of a more resilient and health-promoting microbial ecosystem [48,49]. Functional annotation further revealed significant enrichment in the phosphotransferase system (PTS) and galactose metabolism pathways, suggesting enhanced carbohydrate utilization efficiency and microbial energy turnover. These results are consistent with previous findings observed in tilapia and catfish [50,51], and further expand the intrinsic mechanism underlying microbiome–metabolite symbiosis on the basis of existing research: previous studies have confirmed that a moderately acidic intestinal environment matches the acid-resistant physiological characteristics of lactobacilli [52]. In addition, in vitro screening assays and genomic data demonstrate that *Clostridium butyricum* DMZ-SG06 possesses robust butyrate-synthesizing potential. It is thus inferred that butyrate produced by this strain may reduce intestinal pH and thereby facilitate the proliferation of lactobacilli; reciprocally, metabolites secreted by lactobacilli can enhance the activity of *C. butyricum* populations, forming a positive feedback regulatory loop. This positive feedback loop represents a novel ecological model of “mutual promotion and metabolic symbiosis among microbial communities”, which advances current understanding of interactions between probiotics and the gut microbiota in fish.

Integrating these multi-level findings, we propose a plausible mechanistic model to interpret how *C. butyricum* DMZ-SG06 modulates host physiological functions. This strain exerts a coordinated and synergistic influence on host physiology. At the metabolic level, it enhances carbon source utilization and butyrate synthesis, providing continuous energy for intestinal epithelial maintenance. At the molecular level, elevated transcript levels of TOR were observed, which may contribute to improved antioxidant defenses and immune homeostasis. At the microbiome level, it reconstructs the gut community toward a beneficial configuration that supports SCFA production and pathogen suppression. Collectively, these processes constitute a comprehensive physiological mechanism chain of “butyrate metabolism–signal regulation–microbiome remodeling–health enhancement.”

This study not only validates the biological efficacy of a highly efficient butyrate-producing *C. butyricum* strain in fish but also introduces a mechanistic model integrating metabolic, immune, and microbial dimensions of probiotic action. The findings provide scientific support for the development of functional probiotics as eco-friendly alternatives to antibiotics in aquaculture and establish a theoretical foundation for further exploration of probiotic–host interaction networks regulating intestinal homeostasis and health resilience.

Based on the above, it is also necessary to acknowledge some limitations of the present study. Only one supplementation dose was applied in the feeding trial, so dose–response relationships could not be established. Pathogen challenge assays were not performed, which restricts evidence supporting its use as an antibiotic alternative. Each experimental group contained only three replicate tanks, which may partially limit statistical power. For the T2 group receiving the liquid bacterial suspension (spent fermentation medium), potential vehicle effects originating from liquid-formulation components and residual medium constituents cannot be fully excluded and should be considered when interpreting the corresponding experimental results. Additionally, as this study was performed under laboratory conditions, practical industrial-related parameters such as cost evaluation, shelf-life performance, feed quality control, regulatory approval and large-scale fermentation scalability need further investigation in follow-up applied studies.

## 5. Conclusions

This study identified and characterized *C. butyricum* DMZ-SG06, a butyrate-producing probiotic with strong acid production, stability, and genomic safety. When applied as a feed additive in either powder or liquid form, DMZ-SG06 significantly enhanced growth performance, optimized intestinal morphology, and strengthened antioxidant and immune functions in largemouth bass. These effects were accompanied by favorable modulation of intestinal immune factor expression and gut microbiota composition. The liquid formulation exhibited greater efficacy than the powder, likely due to the synergistic action between live bacterial cells and the released metabolite butyric acid. Collectively, these findings demonstrate that *C. butyricum* DMZ-SG06 promotes host health through coordinated regulation of butyrate metabolism, immune signaling, and microbiota homeostasis. This work provides a scientific foundation for developing *C. butyricum* as a next-generation, eco-friendly probiotic feed additive and offers theoretical guidance for designing composite microbial formulations in sustainable aquaculture. Nevertheless, the results of this study were obtained under laboratory conditions. Future research should validate its performance under practical aquaculture conditions and promote the industrial application of *C. butyricum* DMZ-SG06.

## Figures and Tables

**Table 1:** Composition and nutrient levels of the basal diet for largemouth bass (%).

Item	Content
**Ingredients (air-dried basis)**	
Fish meal	39.49
Soybean meal	19.70
Chicken meal	7.07
Corn gluten meal	10.10
Low gluten wheat flour	14.75
Fish oil	2.53
Soybean Lecithin	2.02
Vitamin and mineral Prem ^1^	2.02
Ca(H_2_PO_4_)_2_	2.02
Choline chloride, 50%	0.30
Total	100.00
**Analyzed nutrient levels** ^2^	
Crude protein	52.31
Crude lipid	13.65
Crude fiber	2.92
Crude ash	15.07
Organic matter ^3^	84.93
Gross energy, MJ/kg	20.49

^1^ Provided per kilogram of diet: vitamin A, 8000 IU; vitamin D_3_, 2000 IU; vitamin E, 100 mg; menadione, 20 mg; thiamine, 16 mg; riboflavin, 16 mg; cobalamin, 0.12 mg; vitamin C, 300 mg; D-calcium pantothenate, 56 mg; niacinamide, 7.6 mg; folic acid, 7.6 mg; D-biotin, 0.16 mg; inositol, 140 mg; FeSO_4_, 36 mg; CuSO_4_·5H_2_O, 9 mg; MnSO_4_·4H_2_O, 0.2 mg; ZnSO_4_, 40 mg; MgSO_4_·7H_2_O, 80 mg; CoCO_3_, 1.4 mg; KI, 1.2 mg; Na_2_SeO_3_, 0.4 mg. ^2^ All are analyzed values on dry matter basis. ^3^ Organic matter (OM) content was calculated as dry matter content minus crude ash content.

**Table 2:** Screening of *C. butyricum* DMZ-SG06 as the optimal butyrate-producing strain.

Item	Groups ^1^			SEM	*p*-Value ^2^		
	DMZ-SG06	DB-02	DB-03		ANOVA	Linear	Quadratic
Butyrate, mg/mL							
2 h	0.29 ^a^	0.21 ^a^	0.21 ^a^	0.037	0.282	0.168	0.437
8 h	0.79 ^a^	0.69 ^a^	0.68 ^a^	0.048	0.310	0.180	0.481
12 h	1.16 ^a^	1.01 ^a^	1.11 ^a^	0.045	0.135	0.453	0.065
16 h	1.38 ^a^	1.17 ^b^	1.32 ^a^	0.032	0.008	0.241	0.003
18 h	1.54 ^a^	1.24 ^b^	1.44 ^c^	0.026	<0.001	0.039	<0.001
24 h	1.65 ^a^	1.33 ^b^	1.52 ^c^	0.025	<0.001	0.013	<0.001

Butyrate = butyric acid; SEM = standard error of the mean. Data are shown as means ± SEM (*n* = 3). ^a,b,c^ Values with different letters in the same row mean significant difference (*p* < 0.05), with highly significant differences indicated at *p* < 0.01 and *p* < 0.001. ^1^ DMZ-SG06: the selected *C. butyricum* strain; DB-02, DB-03: other *C. butyricum* strains preserved in the laboratory. ^2^ ANOVA *p*-values indicate whether differences exist among all groups. One-way ANOVA followed by Tukey’s post hoc test was used for multiple-group comparison. Linear and quadratic polynomial contrasts were performed to evaluate dose–response trends across treatments.

**Table 3:** Effects of *C. butyricum* DMZ-SG06 on growth performance of largemouth bass.

Item	Groups ^1^			SEM	*p*-Value ^2^		
	Con	T1	T2		ANOVA	Linear	Quadratic
WGR, %	192.10 ± 10.20 ^a^	201.40 ± 14.10 ^ab^	213.90 ± 12.30 ^b^	3.566	0.014	0.005	0.727
SGR, %/d	1.79 ± 0.05 ^a^	1.84 ± 0.06 ^ab^	1.91 ± 0.05 ^b^	0.020	0.013	0.005	0.689
FCR ^3^	1.15 ± 0.10	1.10 ± 0.12	1.05 ± 0.11	0.068	0.653	0.384	0.862
FI ^3^, %/d	3.21 ± 0.34	3.34 ± 0.42	3.57 ± 0.41	0.226	0.555	0.303	0.863
SR, %	96.67	98.89	100	-	-	-	-

WGR = weight gain rate; SGR = specific growth rate; FCR = feed conversion ratio; FI = feed intake; SR = survival rate. SEM = standard error of mean. Data are shown as means ± SEM (*n* = 3). ^a,b^ Values with different letters in the same row mean significant difference (*p* < 0.05). Survival rate is presented descriptively and was not subjected to one-way ANOVA. ^1^ Con, T1 and T2 indicate largemouth bass fed a basal diet with 0, 1 g *C. butyricum* DMZ-SG06 powder (5 × 10^8^ CFU/g) per 10 g diet, and 1 mL *C. butyricum* DMZ-SG06 bacterial solution (5 × 10^8^ CFU/mL) per 10 g diet. ^2^ ANOVA *p*-values indicate whether differences exist among all groups. One-way ANOVA followed by Tukey’s post hoc test was used for multiple-group comparison. Linear and quadratic polynomial contrasts were performed to evaluate dose–response trends across treatments. ^3^ Due to the 5 h interval between feeding and siphoning uneaten pellets, partial nutrient leaching of feed may occur, which could introduce potential bias for FI and FCR calculations.

**Table 4:** Intestinal morphology parameters of largemouth bass (μm).

Item	Groups ^1^			SEM	*p*-Value ^2^		
	Con	T1	T2		ANOVA	Linear	Quadratic
Villous height, μm	424.79 ± 15.79 ^a^	475.04 ± 3.90 ^b^	529.87 ± 18.93 ^c^	14.410	0.006	0.002	0.901
Crypt depth, μm	34.75 ± 0.21 ^a^	30.58 ± 0.58 ^b^	28.64 ± 0.26 ^c^	0.383	<0.001	<0.001	0.055
VH/CD	12.23 ± 0.491 ^a^	15.55 ± 0.386 ^b^	18.51 ± 0.815 ^c^	0.593	<0.001	<0.001	0.813

VH/CD = villous height to crypt depth ratio; SEM = standard error of mean. Data are shown as means ± SEM (*n* = 3). ^a,b,c^ Values with different letters in the same row mean significant difference (*p* < 0.05), with highly significant differences indicated at *p* < 0.01 and *p* < 0.001. ^1^ Con, T1 and T2 indicate largemouth bass fed a basal diet with 0, 1 g *C. butyricum* DMZ-SG06 powder (5 × 10^8^ CFU/g) per 10 g diet, and 1 mL *C. butyricum* DMZ-SG06 bacterial solution (5 × 10^8^ CFU/mL) per 10 g diet. ^2^ ANOVA *p*-values indicate whether differences exist among all groups. One-way ANOVA followed by Tukey’s post hoc test was used for multiple-group comparison. Linear and quadratic polynomial contrasts were performed to evaluate dose–response trends across treatments.

**Table 5:** Effects of *C. butyricum* DMZ-SG06 on serum nonspecific immune indexes of largemouth bass.

Item	Groups ^1^			SEM	*p*-Value ^2^		
	Con	T1	T2		ANOVA	Linear	Quadratic
LZM, μg/mL	2.61 ± 0.80 ^a^	5.63 ± 0.87 ^b^	7.87 ± 0.70 ^b^	0.840	0.002	<0.001	0.712
ACP, U/mL	0.02 ± 0.00 ^a^	0.03 ± 0.00 ^b^	0.03 ± 0.00 ^b^	0.002	<0.001	<0.001	0.258
IgM, μg/mL	9.22 ± 0.62 ^a^	11.78 ± 1.36 ^b^	14.60 ± 1.62 ^c^	0.614	<0.001	<0.001	0.606

LZM = lysozyme; ACP = acid phosphatase; IgM = immunoglobulin M; SEM = standard error of mean. Data are shown as means ± SEM (*n* = 3). ^a,b,c^ Values with different letters in the same row mean significant difference (*p* < 0.05), with highly significant differences indicated at *p* < 0.01 and *p* < 0.001. ^1^ Con, T1 and T2 indicate largemouth bass fed a basal diet with 0, 1 g *C. butyricum* DMZ-SG06 powder (5 × 10^8^ CFU/g) per 10 g diet, and 1 mL *C. butyricum* DMZ-SG06 bacterial solution (5 × 10^8^ CFU/mL) per 10 g diet. ^2^ ANOVA *p*-values indicate whether differences exist among all groups. One-way ANOVA followed by Tukey’s post hoc test was used for multiple-group comparison. Linear and quadratic polynomial contrasts were performed to evaluate dose–response trends across treatments.

**Table 6:** Effects of *C. butyricum* DMZ-SG06 on serum antioxidant capacity of largemouth bass.

Item	Groups ^1^			SEM	*p*-Value ^2^		
	Con	T1	T2		ANOVA	Linear	Quadratic
SOD, U/mL	3.15 ± 0.55 ^a^	6.98 ± 1.23 ^b^	11.22 ± 1.68 ^c^	1.242	0.001	<0.001	0.894
CAT, U/mL	75.42 ± 1.34 ^a^	77.88 ± 1.56 ^a^	83.10 ± 1.16 ^b^	1.594	0.012	0.004	0.489
GSH-Px, U/L	544.41 ± 6.00 ^a^	545.48 ± 9.08 ^a^	580.21 ± 10.03 ^b^	8.547	0.015	0.010	0.129
T-AOC, μmol/mL	0.05 ± 0.01 ^a^	0.18 ± 0.03 ^b^	0.23 ± 0.03 ^b^	0.026	<0.001	<0.001	0.246
MDA, nmol/mL	1.76 ± 0.09 ^a^	1.41 ± 0.08 ^b^	0.93 ± 0.03 ^c^	0.071	<0.001	<0.001	0.476

SOD = superoxide dismutase; CAT = catalase; GSH-Px = glutathione peroxidase; T-AOC = total antioxidant capacity; MDA = malondialdehyde; SEM = standard error of mean. Data are shown as means ± SEM (*n* = 3). ^a,b,c^ Values with different letters in the same row mean significant difference (*p* < 0.05), with highly significant differences indicated at *p* < 0.01 and *p* < 0.001. ^1^ Con, T1 and T2 indicate largemouth bass fed a basal diet with 0, 1 g *C. butyricum* DMZ-SG06 powder (5 × 10^8^ CFU/g) per 10 g diet, and 1 mL *C. butyricum* DMZ-SG06 bacterial solution (5 × 10^8^ CFU/mL) per 10 g diet. ^2^ ANOVA *p*-values indicate whether differences exist among all groups. One-way ANOVA followed by Tukey’s post hoc test was used for multiple-group comparison. Linear and quadratic polynomial contrasts were performed to evaluate dose–response trends across treatments.

**Table 7:** Effects of *C. butyricum* DMZ-SG06 on intestinal inflammatory cytokines.

Item	Groups ^1^			SEM	*p*-Value ^2^		
	Con	T1	T2		ANOVA	Linear	Quadratic
TNF-α, pg/g	178.67 ± 7.80 ^a^	150.52 ± 9.91 ^a^	128.57 ± 4.97 ^b^	7.890	0.002	<0.001	0.753
IL-1β, pg/g	260.08 ± 8.61 ^a^	213.62 ± 12.22 ^b^	166.55 ± 10.87 ^c^	10.470	<0.001	<0.001	0.982
IL-10, pg/g	244.31 ± 7.57 ^a^	306.87 ± 13.50 ^b^	321.89 ± 15.03 ^b^	12.450	0.001	<0.001	0.140

TNF-α = tumor necrosis factor α; IL-1β = interleukin 1β; IL-10 = interleukin 10; SEM = standard error of mean. Data are shown as means ± SEM (*n* = 3). ^a,b,c^ Values with different letters in the same row mean significant difference (*p* < 0.05), with highly significant differences indicated at *p* < 0.01 and *p* < 0.001. ^1^ Con, T1 and T2 indicate largemouth bass fed a basal diet with 0, 1 g *C. butyricum* DMZ-SG06 powder (5 × 10^8^ CFU/g) per 10 g diet, and 1 mL *C. butyricum* DMZ-SG06 bacterial solution (5 × 10^8^ CFU/mL) per 10 g diet. ^2^ ANOVA *p*-values indicate whether differences exist among all groups. One-way ANOVA followed by Tukey’s post hoc test was used for multiple-group comparison. Linear and quadratic polynomial contrasts were performed to evaluate dose–response trends across treatments.

## Data Availability

The genome of strain DMZ-SG06 has been submitted to NCBI GenBank (BioProject: PRJNA1513492; BioSample: SAMN62489699). All supplementary data supporting the conclusions are provided in the Supplementary Materials of this article.

## Supplementary Materials

The following supporting information can be downloaded at: https://www.mdpi.com/article/10.3390/microorganisms14091931/s1, Figure S1: Characterization of *C. butyricum* DMZ-SG06; Figure S2: Barplot of species-functional contribution analysis; Table S1: Average nucleotide identity (ANI) between *Clostridium butyricum* DMZ-SG06 and the type strain *Clostridium butyricum* DSM 10702; Table S2: Digital DNA–DNA hybridization (dDDH) between *Clostridium butyricum* DMZ-SG06 and the type strain *Clostridium butyricum* DSM 10702; Table S3: Ct values of reference gene EF-1α across different treatment groups; Table S4: Real-time quantitative PCR primer pair sequences; Table S5: Screening of *C. butyricum* DMZ-SG06 based on spore germination rate; Table S6: Screening of *C. butyricum* DMZ-SG06 based on total acid production; Table S7: Screening of *C. butyricum* DMZ-SG06 based on storage survival stability; Table S8: Acid tolerance characterization of *C. butyricum* DMZ-SG06; Table S9: Bile salt tolerance characterization of *C. butyricum* DMZ-SG06; Table S10: Storage stability characterization of *C. butyricum* DMZ-SG06 powdered cultures; Table S11: Genome features of DMZ-SG06; Table S12: Antibiotic Resistance Genes and Virulence Factors in Strain DMZ-SG06; Table S13: Genes related to butyrate biosynthesis and energy metabolism in strain DMZ-SG06; Table S14: Exact *p*-values for pairwise comparisons of intestinal cytokine mRNA expression.

## Abbreviations

ACP	Acid phosphatase
ANOSIM	Analysis of similarities
CAZy	Carbohydrate-Active enZYmes
CAT	Catalase
COG	Clusters of Orthologous Groups
ELISA	Enzyme-linked immunosorbent assay
FCR	Feed conversion ratio
FI	Feed intake
GSH-Px	Glutathione peroxidase
GHs	Glycoside hydrolases
GTs	Glycosyltransferases
GO	Gene Ontology
GE	Gross energy
H&E	Hematoxylin and Eosin
HSP70	Heat shock protein 70
IgM	Immunoglobulin M
IL-1β	Interleukin-1β
IL-10	Interleukin-10
IL-15	Interleukin-15
KEGG	Kyoto Encyclopedia of Genes and Genomes
LDA	Linear discriminant analysis
LZM	Lysozyme
MDA	Malondialdehyde
mTOR	Mammalian target of rapamycin
NF-κB	Nuclear factor-κB
NMDS	Non-metric multidimensional scaling
OM	Organic matter
PCA	Principal component analysis
PCoA	Principal coordinate analysis
qRT-PCR	Quantitative real-time polymerase chain reaction
SCFAs	Short-chain fatty acids
SGR	Specific growth rate
SOD	Superoxide dismutase
AOC	Total antioxidant capacity
TGF-β	Transforming growth factor-β
TNF-α	Tumor necrosis factor-α
TSC	Tryptose Sulfite Cycloserine Agar Base
VH/CD	Villus height/crypt depth
WGR	Weight gain rate
