# Supplementary material for: A Butyrate-Producing Probiotic, Clostridium butyricum DMZ-SG06, Improves Growth Performance, Immune Function and Gut Microbiota in Largemouth Bass (Micropterus salmoides)
Source: Microorganisms. 2026 Sep 1;14(9):1931. doi: 10.3390/microorganisms14091931 (PMC13609853; doi:10.3390/microorganisms14091931)
Supplement: Supplementary file 1 [file microorganisms-14-01931-s001.zip › microorganisms-4452987-supplementary.pdf]

Table S1 Average nucleotide identity (ANI) between *Clostridium butyricum* DMZ-SG06 and the type strain *Clostridium butyricum* DSM 10702.

Metric	Value
OrthoANI value (%)	99.00
Genome A (DMZ-SG06) length (bp)	4,624,680
Genome B (DSM 10702) length (bp)	4,504,320
Average aligned length (bp)	2,893,329
Genome A (DMZ-SG06) coverage (%)	62.56
Genome B (DSM 10702) coverage (%)	64.23

Table S2 Digital DNA–DNA hybridization (dDDH) between *Clostridium butyricum* DMZ-SG06 and the type strain *Clostridium butyricum* DSM 10702.

Metric	Value
dDDH value (Formula 2, %)	91.80
Confidence interval (%)	89.7 – 93.5
Probability DDH ≥ 70% (%)	96.36
G+C content difference (%)	0.22

Table S3 Ct values of reference gene EF-1α (EF-1α) across different treatment groups.

Group	Ct value (mean ± SEM)
Con	20.42 ± 0.06
T1	20.33 ± 0.07
T2	20.22 ± 0.14

One-way ANOVA, P > 0.05. No significant difference was observed among groups.

Table S4 Real-time quantitative PCR primer pair sequences.

Genes	Forward (5´to 3´)	Reverse (5´to 3´)
EF-1α	GGCTGGTATCTCCAAGAACG	GTCTCCAGCATGTTGTCWCC
TNF-α	CTTCGTCTACAGCCAGGCATCG	TTTGGCACACCGACCTCACC
IL-1β	CGTGACTGACAGCAAAAAGAGG	GATGCCCAGAGCCACAGTTC
IL-10	CGGCACAGAAATCCCAGAGC	CAGCAGGCTCACAAAATAAACATCT
HSP70	CAGTGATGAAGACAAGCAGAAGA	GCCACCAGCACTCTGATACA
IL-15	AGCGTCAGATTTCTCAATGGTGT	CGTGACTGACAGCAAAAAGAGG
TGF-β	GCTCAAAGAGAGCGAGGATG	TCCTCTACCATTCGCAATCC
TOR	TCAGGACCTCTTCTCATTGG	CCTCTCCCACCATGTTTCTCT

EF-1α= elongation factor 1 α; TNF-α = tumor necrosis factor α; IL-1β = interleukin 1β; IL-10 = interleukin 10;HSP70 = heatshock protein 70; IL-15 = interleukin 15; TGF-β = transforming growth factor β; TOR = target of rapamycin.

Table S5 Screening of *C. butyricum* DMZ-SG06 based on spore germination rate.

Item	Groups[1]			SEM	*P*-value[2]		
	DMZ-SG06	DB-02	DB-03		ANOVA	Linear	Quadratic
**Absorbance,OD_{600}**							
0h	0.20[a]	0.18[a]	0.18[a]	0.041	0.888	0.709	0.776
2h	0.43[a]	0.28[b]	0.30[b]	0.026	0.020	0.019	0.049
4h	1.50[a]	1.22[b]	1.32[b]	0.037	0.006	0.015	0.007
6h	1.86[a]	1.75[a]	1.71[a]	0.048	0.162	0.074	0.589
8h	1.91[a]	1.78[b]	1.75[b]	0.032	0.025	0.015	0.121
10h	1.95[a]	1.83[b]	1.78[b]	0.024	0.012	0.005	0.499
12h	2.03[a]	1.85[a]	1.80[a]	0.063	0.087	0.042	0.379

Absorbance = Optical density at 600 nm (OD_{600}); SEM = Standard error of the mean.

Data were shown as means ± SEM (n=3). [a,b] Values with different superscript letters in the same row mean significant difference ($P < 0.05$), with highly significant differences indicated at $P < 0.01$ and $P < 0.001$.

[1]DMZ-SG06: The selected *C. butyricum* strain; DB-02, DB-03: Other *C. butyricum* strains preserved in the laboratory.

[2]ANOVA P-values indicate whether differences exist among strains. Linear and quadratic P-values indicate trends in spore germination over time. All analyses used one-way ANOVA with Tukey's post-hoc test.

Table S6 Screening of *C. butyricum* DMZ-SG06 based on total acid production.

Item	Groups[1]			SEM	*P*-value[2]		
	DMZ-SG06	DB-02	DB-03		ANOVA	Linear	Quadratic
**Total acid,%**							
2h	0.44^{a}	0.41^{a}	0.41^{a}	0.026	0.680	0.466	0.656
4h	0.65^{a}	0.60^{a}	0.46^{b}	0.032	0.017	0.008	0.226
6h	0.72^{a}	0.69^{a}	0.52^{b}	0.018	<0.001	<0.001	0.021
8h	0.76^{a}	0.72^{a}	0.63^{a}	0.037	0.118	0.050	0.623
10h	0.82^{a}	0.77^{a}	0.68^{a}	0.041	0.120	0.050	0.680
12h	0.83^{a}	0.79^{a}	0.73^{a}	0.031	0.224	0.098	0.867

Total acid = The total concentration of organic acids produced; SEM = Standard error of the mean. Data were shown as means ± SEM (n=3). ^{a,b} Values with different superscript letters in the same row mean significant difference ($P < 0.05$), with highly significant differences indicated at $P < 0.01$ and $P < 0.001$.

[1]DMZ-SG06: The selected *C. butyricum* strain; DB-02, DB-03: Other *C. butyricum* strains preserved in the laboratory.

[2]ANOVA P-values indicate whether differences exist among strains. Linear and quadratic P-values indicate trends in total acid production over time. All analyses used one-way ANOVA with Tukey's post-hoc test.

Table S7 Screening of *C. butyricum* DMZ-SG06 based on storage survival stability.

Item	Groups[1]			SEM	*P*-value[2]		
	DMZ-SG06	DB-02	DB-03		ANOVA	Linear	Quadratic
**90-Day Ambient Storage Survival,%**							
	92.90[a]	85.17[ab]	76.56[b]	2.328	0.007	0.003	0.884
**10-Day 40℃ Storage Survival,%**							
	76.04[a]	70.85[b]	55.43[c]	2.080	0.001	<0.001	0.092

SEM = Standard error of the mean.

Data were shown as means ± SEM (n=3). [a,b,c] Values with different superscript letters in the same row mean significant difference ($P < 0.05$), with highly significant differences indicated at $P < 0.01$ and $P < 0.001$.

[1]DMZ-SG06: The selected *C. butyricum* strain; DB-02, DB-03: Other *C. butyricum* strains preserved in the laboratory.

[2]ANOVA P-values indicate whether differences exist among strains. Linear and quadratic P-values indicate differences in survival rate across storage conditions. All analyses used one-way ANOVA with Tukey's post-hoc test.

Table S8 Acid tolerance characterization of *C. butyricum* DMZ-SG06.

Item	Groups[1]		SEM	*P*-value[2]
	Control	PH=2.0		ANOVA
**Acid tolerance, $\log_{10}$ CFU/g**				
0h	11.07[a]	10.76[a]	0.246	0.410
1h	11.07[a]	10.75[a]	0.247	0.416
2h	11.09[a]	11.08[a]	0.018	0.717

Acid tolerance = Viable cell count ($\log_{10}$ CFU/g) after incubation at pH=2.0; SEM = Standard error of the mean.

Data were shown as means ± SEM (n=3). [a,b] Values with different superscript letters in the same row indicate significant difference between groups ($P < 0.05$), with highly significant differences indicated at $P < 0.01$ and $P < 0.001$.

[1]Control: *C. butyricum* DMZ-SG06 cultured under neutral conditions; pH=2.0: *C. butyricum* DMZ-SG06 incubated at pH=2.0.

[2]ANOVA P-values indicate whether differences exist between the control and acid-treated groups. Linear and quadratic P-values indicate changes in viable cell count over time. All analyses used one-way ANOVA with Tukey's post-hoc test.

Table S9 Bile salt tolerance characterization of *C. butyricum* DMZ-SG06.

Item	Groups[1]			SEM	*P*-value[2]		
	Control	0.25%	0.5%		ANOVA	Linear	Quadratic
**Bile tolerance,OD_{600}**							
0h	0.20^{a}	0.09^{b}	0.08^{b}	0.019	0.016	0.010	0.089
2h	0.35^{a}	0.38^{a}	0.16^{b}	0.036	0.002	0.003	0.010
4h	1.39^{a}	1.19^{b}	0.99^{c}	0.051	<0.001	<0.001	0.979
6h	1.71^{a}	1.63^{ab}	1.53^{b}	0.032	0.016	0.006	0.804
8h	1.71^{a}	1.73^{a}	1.62^{b}	0.018	0.007	0.009	0.020
10h	1.72^{a}	1.71^{a}	1.69^{a}	0.006	0.224	0.114	0.526
12h	1.72^{a}	1.72^{a}	1.70^{a}	0.008	0.256	0.154	0.409

Bile tolerance = Bacterial growth estimated by optical density at 600 nm (OD_{600}); SEM = Standard error of the mean.

Data were shown as means ± SEM (n=3). [a,b] Values with different superscript letters in the same row indicate significant difference among groups ($P < 0.05$), with highly significant differences indicated at $P < 0.01$ and $P < 0.001$.

[1]Control: *C. butyricum* DMZ-SG06 cultured in unsupplemented medium; 0.25%: *C. butyricum* DMZ-SG06 cultured in medium supplemented with 0.25% bile salts; 0.5%: *C. butyricum* DMZ-SG06 cultured in medium supplemented with 0.5% bile salts.

[2]ANOVA P-values indicate whether differences exist among the control and bile salt-supplemented groups. Linear and quadratic P-values indicate trends in bacterial growth over time. All analyses used one-way ANOVA with Tukey's post-hoc test.

Table S10 Storage stability characterization of *C. butyricum* DMZ-SG06 powdered cultures.

Item	Groups[1]		SEM	*P*-value[2]
	1.0×10^9CFU/g	5.0×10^8CFU/g		ANOVA
**Survival,%**				
0d	100.00[a]	100.03[a]	0.514	0.965
30d	98.07[a]	96.00[a]	0.607	0.074
60d	85.10[a]	88.10[b]	0.666	0.033
90d	77.60[a]	81.03[b]	0.582	0.014
120d	74.67[a]	75.83[a]	0.951	0.434

Survival = Percentage of viable *C. butyricum* cells remaining in the powdered culture; SEM = Standard error of the mean.

Data were shown as means ± SEM (n=3). [a,b] Values with different superscript letters in the same row indicate significant difference between groups ($P < 0.05$), with highly significant differences indicated at $P < 0.01$ and $P < 0.001$.

[1]1.0×10^9 CFU/g: Powdered culture prepared at 1.0×10^9 CFU/g; 5.0×10^8 CFU/g: Powdered culture prepared at 5.0×10^8 CFU/g.

[2]ANOVA P-values indicate whether differences exist between the two powdered culture concentrations. Linear and quadratic P-values indicate changes in survival rate over storage time. All analyses used one-way ANOVA with Tukey's post-hoc test.

Table S11 Genome features of DMZ-SG06

Features	Value
Genome size (bp)	4,625,003
Chromosome	1
Topology	Circular
G+C content (%)	28.71
Protein-coding genes (CDS)	4,210
Gene total length (bp)	3,883,998
Plasmid length (bp)	769,297
Gene Len/genome (%)	83.98
Intergenetic region length (bp)	741,005
Intergenetic length/genome (%)	16.02
rRNA (5S rRNA, 16S rRNA, 23S rRNA)	10, 10, 10
tRNA	87
sRNA	3
Prophage	9
CRISPR	6
NR annotation	4,204
Swiss-Prot annotation	1,458
COG annotation	2,852
GO annotation	2,549
KEGG annotation	2,306
CAZy annotation	204

Table S12 Antibiotic Resistance Genes and Virulence Factors in Strain DMZ-SG06

a. Antibiotic Resistance Genes

Gene ID	Database Source	Identity (%)	E-value	Resistance Gene Name	Associated Antibiotics	Risk Level	Annotation
DMZ-SG06 GL002587	CARD	100	2.2e-278	emrA	Fluoroquinolones (Levofloxacin, Moxifloxacin, Ciprofloxacin)	Low	Encodes a major facilitator superfamily (MFS) efflux pump; mediates resistance by extruding antibiotics to reduce intracellular concentration. Identified with strict cutoffs (Identity≥80%, Coverage≥70%) ensuring high confidence.

b. Virulence Factors

Gene ID	Database Source	Identity (%)	E-value	Virulence Factor Name	Toxicity Trait	Risk Level	Annotation
DMZ-SG06 GL000029	VFDB	78.6	1.1e-99	Hemolysin III	Erythrocyte membrane disruption activity	Low	Channel protein belonging to the hemolysin III family (CVF417); no evidence of strong pathogenicity; hemolytic activity requires experimental validation.
DMZ-SG06 GL001362	VFDB	84.4	2.4e-128	Hemolysin A	Erythrocyte membrane disruption activity	Low	Homologous to *Clostridium beijerinckii* hemolysin A; strain-specific activity with no reports of high pathogenicity.
DMZ-SG06 GL001652	VFDB	54.3	3.1e-127	Putative hemolysin (hlyB homolog)	Erythrocyte membrane disruption activity	Low	Homolog of *Clostridium perfringens* hlyB; low sequence conservation indicates weak toxicity.
DMZ-SG06 GL002271	VFDB	45.3	9.1e-77	Ornithine formyltransferase	Phaseolotoxin-like (ornithine formyltransferase)	Low	Associated with plant toxin phaseolotoxin metabolism; lacks a complete carbamate metabolic pathway, precluding functional toxin production.

Table S13 Genes related to butyrate biosynthesis and energy metabolism in strain DMZ-SG06

Functional Category	Key Gene/Enzyme	KEGG Pathway Name	KEGG Pathway ID	KO ID	Gene ID (Strain DMZ-SG06)	Annotation Note
Butyrate biosynthesis (Acetyl-CoA pathway)	Butyryl-CoA:acetate CoA-transferase	Butanoate metabolism	ko00650	K01034	DMZ-SG06_gene_0872; DMZ-SG06_gene_1245	Core enzyme for butyrate synthesis; catalyzes the conversion of butyryl-CoA to butyrate using acetate as an acceptor
Butyrate biosynthesis (Acetyl-CoA pathway)	Butyrate kinase	Butanoate metabolism	ko00650	K00929	DMZ-SG06_gene_0518; DMZ-SG06_gene_1693	Key enzyme for butyrate synthesis; phosphorylates butyrate to form butyryl-phosphate in the final step of butyrate production
Energy metabolism (Redox process)	Oxidoreductases	Oxidative phosphorylation; Carbon metabolism	ko00190; ko01200	K00123; K00149	DMZ-SG06_gene_0231; DMZ-SG06_gene_0987; DMZ-SG06_gene_1562	Involved in electron transfer and redox reactions; supports efficient energy generation by mediating proton gradient formation
Energy metabolism (ATP synthesis)	ATP synthase complex	Oxidative phosphorylation	ko00190	K02111; K02112	DMZ-SG06_gene_0345; DMZ-SG06_gene_0789; DMZ-SG06_gene_1123	Catalyzes ATP synthesis using the proton gradient; enhances adaptability to hypoxic intestinal environments by optimizing energy utilization

Table S14 Exact P-values for pairwise comparisons of intestinal cytokine mRNA expression

Gene	Con vs T1	Con vs T2	T1 vs T2
TNF-α	—	P = 0.0010	—
IL-1β	P = 0.0018	P < 0.0001	—
IL-10	—	P < 0.0001	P = 0.0025
HSP70	—	—	P = 0.0031
IL-15	P = 0.0013	P < 0.0001	P = 0.0169
TGF-β	—	P < 0.0001	P < 0.0001
TOR	—	P < 0.0001	P = 0.0004

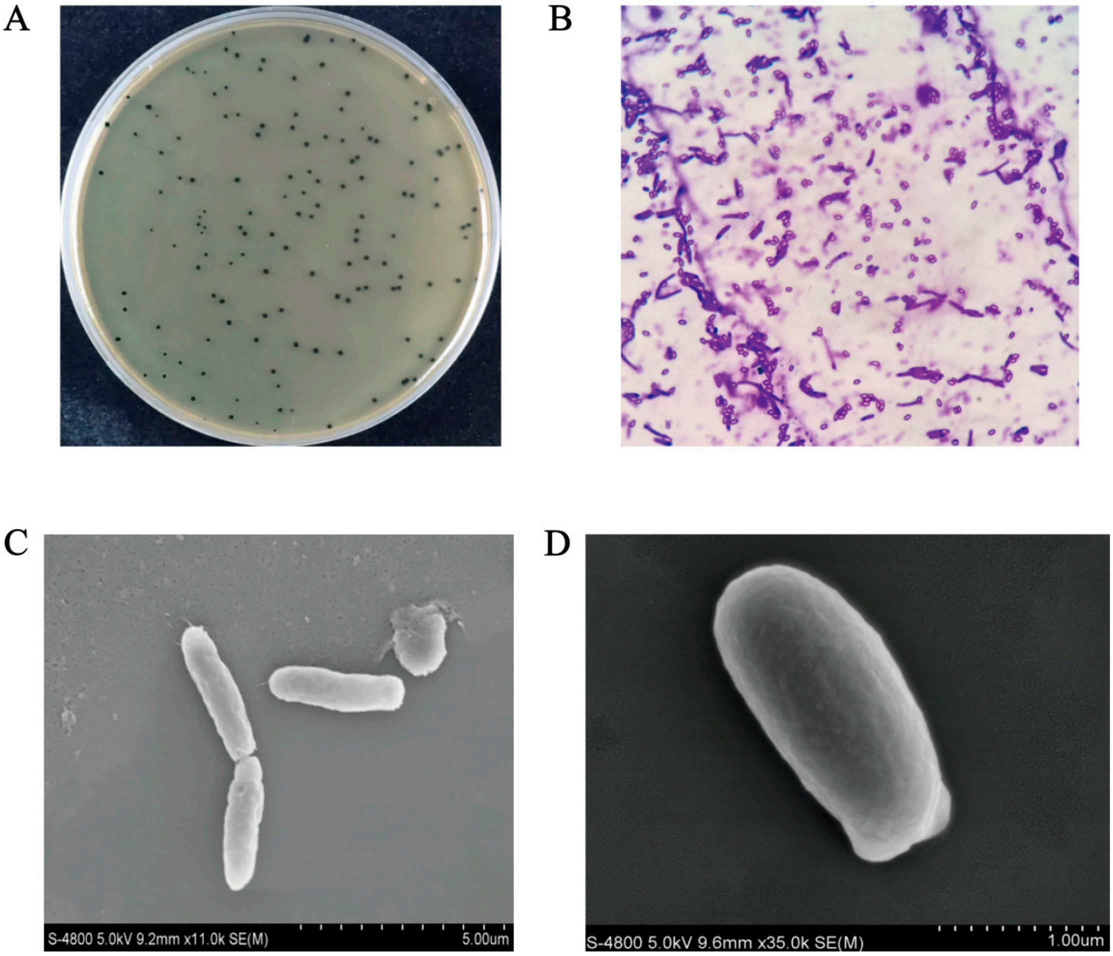

Figure. S1 Characterization of *C. butyricum* DMZ-SG06. (A) Colony morphology on TSC solid medium. (B) Gram staining image under light microscope. (C) Scanning electron microscope (SEM) image of vegetative cells. (D) SEM image of spores.

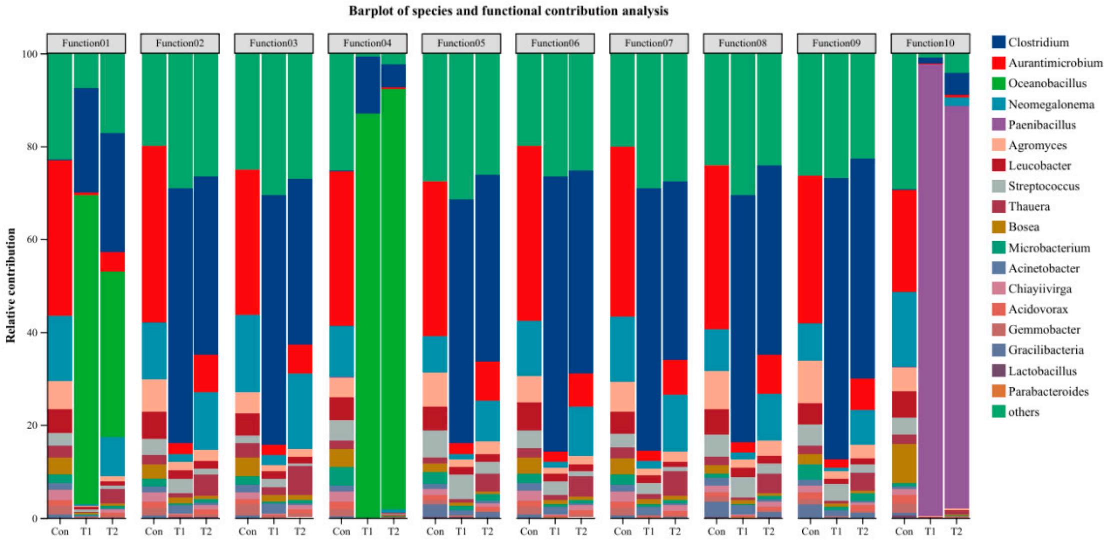

Figure. S2 Barplot of species-functional contribution analysis. This analysis maps functional gene reads to their taxonomic origins and quantifies the relative proportion of each microbial taxon contributing to total gene abundance of a given function. Functional categories include: Function 01: Metabolic pathways; Function 02: Biosynthesis of secondary metabolites; Function 03: Microbial metabolism in diverse environments; Function 04: Cysteine and methionine metabolism; Function 05: DNA replication; Function 06: Biosynthesis of amino acids; Function 07: Biosynthesis of cofactors; Function 08: Homologous recombination; Function 09: Mismatch repair; Function 10: Quorum sensing. Each stacked bar sums to 100%, representing the relative contribution of different microbial genera to the corresponding functional category within each sample (Con, T1, T2).
